# How Marine Megabenthos Fauna Responds to River Discharge and Artificial Flood in Large River Estuary

**DOI:** 10.1002/ece3.70755

**Published:** 2025-01-08

**Authors:** Debin Sun, Qinglu Fu, Jiao Wang, Linlin Chen, Jing Chen, Yilin Wang, Baoquan Li

**Affiliations:** ^1^ CAS Key Laboratory of Coastal Environmental Processes and Ecological Remediation Yantai Institute of Coastal Zone Research, Chinese Academy of Sciences Yantai China; ^2^ University of Chinese Academy of Sciences Beijing China; ^3^ School of Ocean Yantai University Yantai China; ^4^ Center for Ocean Mega‐Science Chinese Academy of Sciences Qingdao China; ^5^ Faculty of Forestry University of British Columbia Vancouver Canada

**Keywords:** artificial flood, biodiversity, community stability, community structure, estuarine ecology, megabenthos

## Abstract

Estuaries are ecologically sensitive areas influenced by river regulation. Knowledge of how marine megabenthos responds to river regulation and artificial flooding events remains limited. The study aims to provide a comprehensive understanding of the impacts of river regulation on marine megabenthic fauna. The oceanographic investigations were conducted at the Yellow River Estuary and its adjacent area during three distinct periods of river discharge, that is, spring low‐flow (IA), summer low‐flow (IB), and artificial flood (IC) periods. Samples of megabenthos and 14 mainly environmental parameters were investigated during different periods. The comprehensive indices of biodiversity (*C‐diversity*), phylogeny (*C‐phyl*), and stability (*C‐stability*) were synthesized from 24 basic indices to characterize the overall traits of megabenthos faunas. All 210 species were collected during three periods, belonging to 16 classes and 51 orders. The typical estuarine communities were mainly distributed in the key area within 30 km of the estuary during the low‐flow periods. The estuarine community presented with low biodiversity (*C‐diversity* = 0.15) but high stability (*C‐stability* = 0.64) during the IA period, followed by an increase in biodiversity during the IB period (*C‐diversity* = 0.35, *C‐phyl* = 0.69). The community underwent dramatic changes during the IC period, which bifurcated into distinct northern and southern groups. The northern community maintained high biodiversity and homeostasis (*C‐stability* = 0.60), whereas the southern stability decreased sharply (0.11). On the whole, the artificial flood reshaped the geographical distribution of megabenthic fauna in the estuarine area within 20 days, mainly as a result of the dramatic changes in seawater salinity and nutrient structure caused by flow pulse. An eco‐friendly discharge management of the Yellow River is needed to mitigate the impact.

## Graphical abstract

The artificial flood reshaped the geographical distribution of megabenthic fauna in the estuarine area in a short time, mainly as a result of the dramatic changes in seawater salinity and nutrients caused by flow pulse. An eco‐friendly discharge management of the Yellow River is needed to weaken the impact.

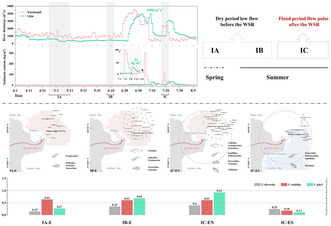

## 1 Introduction

**FIGURE 1:**
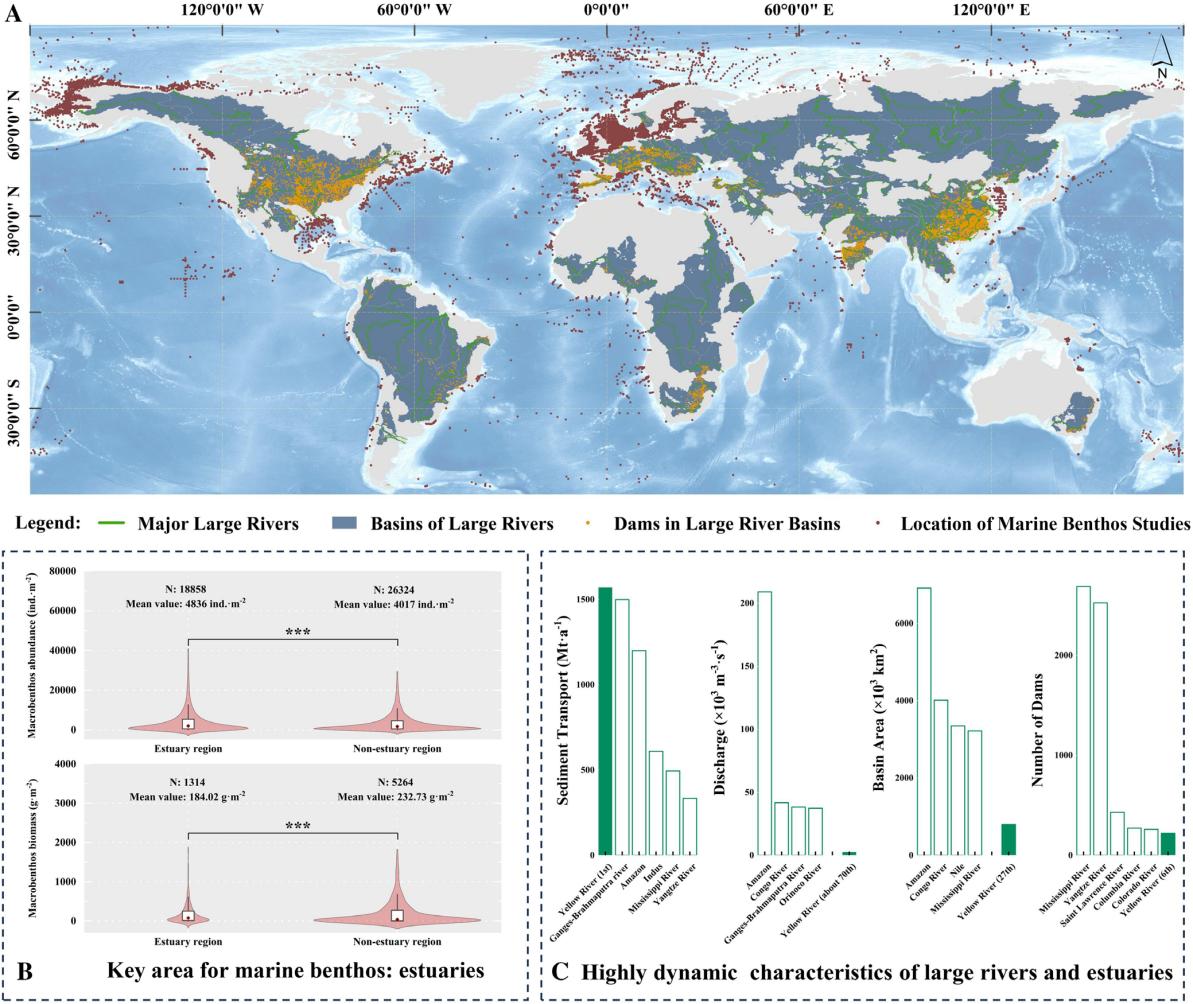
Statistical analysis of global marine benthos and the characteristics of large rivers worldwide. (A) Sampling sites of global marine benthos for statistical analysis. (B) Comparison of megabenthic abundance and biomass in estuary and nonestuary regions, respectively. ***Denotes a significant difference between estuarine areas and other areas (*p* < 0.001). (C) River discharge, sediment transport, and watershed characteristics of global major large river.

Estuaries, serving as transitional areas connecting terrestrial, riverine, and marine ecosystems, exhibit a unique blend of biotic and abiotic factors that contribute to a high productivity and biodiversity (Kaiser 1998; Cloern et al. 2016). River discharge transports substantial amounts of materials to estuarine regions, including freshwater, sediment, and nutrients, playing a crucial role in facilitating the movement, reproduction, and spawning of marine organisms (Griffiths et al. 2017; Hutchins and Capone 2022). However, regulations of river flow and dam constructions have been implemented in numerous large river basins, leading to alterations in the natural flow regime. The partial or complete impoundment, chronic low flows, and drastic peak flows inevitably result in discharge and sediment deposition alterations into the estuary. This has become a worldwide phenomenon, especially in large rivers with wide drainage spans (Figure 1) (Zarfl et al. 2015). Artificial flooding, in particular, can dramatically alter the estuarine ecology in the short term and become an important disturbance event in the estuary (Rosenberg, Mccully, and Pringle 2000; Ezcurra et al. 2019).

Marine benthos is crucial in promoting biogeochemical processes, providing food resources for higher trophic levels and maintaining the structural integrity of aquatic ecosystems (Szczepanek et al. 2021; Silberberger et al. 2024). Given their ecological importance and sensitivity to changes in habitat conditions, marine benthos have been extensively studied as indicators of ecological state in response to natural and anthropogenic impacts (Li et al. 2017). Global meta‐analyses have demonstrated that estuarine areas exhibit distinct patterns of marine benthos, often characterized by higher abundance but lower biomass compared to other marine habitats (*p* < 0.01) (Figure 1). These estuarine areas are typically dominated by small‐sized, fast‐growing, and highly reproductive species, and the community exhibits characteristics of high responsiveness, high vulnerability, and low stability in response to external pressures. More serious, nonrhythmic river discharge further disturbs this vulnerable habitat, affecting the biodiversity and functional roles of resident megabenthos within estuarine ecosystems (Yi et al. 2021; Lam‐Gordillo, Baring, and Dittmann 2020; Liu, Qiao, et al. 2020; Liu, Yi, et al. 2020). Understanding the response characteristics of marine megebenthic fauna to artificial flooding and river regulation is important for ecological maintenance.

Several studies showed that the sudden increase in freshwater and sediment discharge resulting from artificial floods subsequent to prolonged low flow can disturb marine benthos through modifications in substrate composition and the burial of benthic organisms beneath sediment layers (Li et al. 2023). Increased sedimentation resulting from high discharge events can dramatically impact benthic habitats, such as changes in salinity can alter species composition and community structures (Yang et al. 2023). Short‐term spikes in nutrient levels may temporarily boost primary productivity but can also lead to hypoxic conditions detrimental to aerobic benthic fauna (Zhang et al. 2023; Li et al. 2024). The composition and structure of the megabenthic community, especially mollusks and demersal fishes, are significantly altered, and the number of opportunistic species increased (Ren et al. 2016; Li et al. 2020; Liu, Qiao, et al. 2020; Liu, Yi, et al. 2020). Conversely, extremely low‐flow conditions may reduce habitat quality and limit food availability for organisms (Yi, Zhao, et al. 2023). Low discharge during the spawning period and high discharge during the flood period were not conducive to the secondary productivity of the estuarine ecosystem (Yi et al. 2021).

The Yellow River annually contributes over 200 × 10^8^ m^3^ of freshwater and over 15 × 10^8^ tons of sediment into the western Pacific (Ren et al. 2002; http://www.mwr.gov.cn/sj/). There are more than 250 dams in the basin, and the freshwater flux and sediment flux in the estuary are highly dynamic due to the influence of river regulation (Figure 1). As a core process of the Water Sediment Discharge Regulation Scheme (WSRS), artificial floods are transported over 60% of the annual sediment load and 40% of the annual nutrient flux to the estuary within 20 days after chronic low traffic (Li and Sheng 2011; Li et al. 2020). Influenced by the river regulation, the river discharge exhibited distinct two periods, for example, dry periods (mainly in spring and summer before the artificial flood) and human‐controlled flood periods (mainly in July), leading the marine conditions and organisms in this region sensitive to the changes (Zhang et al. 2021; Yi, Gao, et al. 2023). The controlled process of the WSRS is a significant disturbance event that influences the adjacent marine ecological process, involving various organisms (e.g., phytoplankton, zooplankton, ichthyoplankton, and macrobenthos) (Ren et al. 2016; Zhang et al. 2021; Liu et al. 2023; Yang et al. 2023). Considering the widely observed adverse ecological effects of river regulation in estuarine systems worldwide, the insights gained from the river regulation in the Yellow River would be of significant interest to provide an exemplary attempt to meet the “environmental flow requirements” (Flow alteration imperils freshwater and estuarine ecosystems; Brisbane Declaration 2007) and the mission of the “Mega‐Delta Programme” (The Intergovernmental Oceanographic Commission of UNESCO; 2021) (Arthington et al. 2018; Wu et al. 2024).

This study systematically evaluates the characteristic responses of marine megabenthos (biodiversity, phylogenetic diversity, and community stability) in the estuary across different river discharge periods, particularly the artificial flooding period. The study aims to provide a comprehensive understanding of the impacts of river regulation on marine benthos fauna and contribute to the rational flux management associated with the ecological well‐being of the estuary.

## 2 Methods

### 2.1 Study Area and Sampling Sites

**FIGURE 2:**
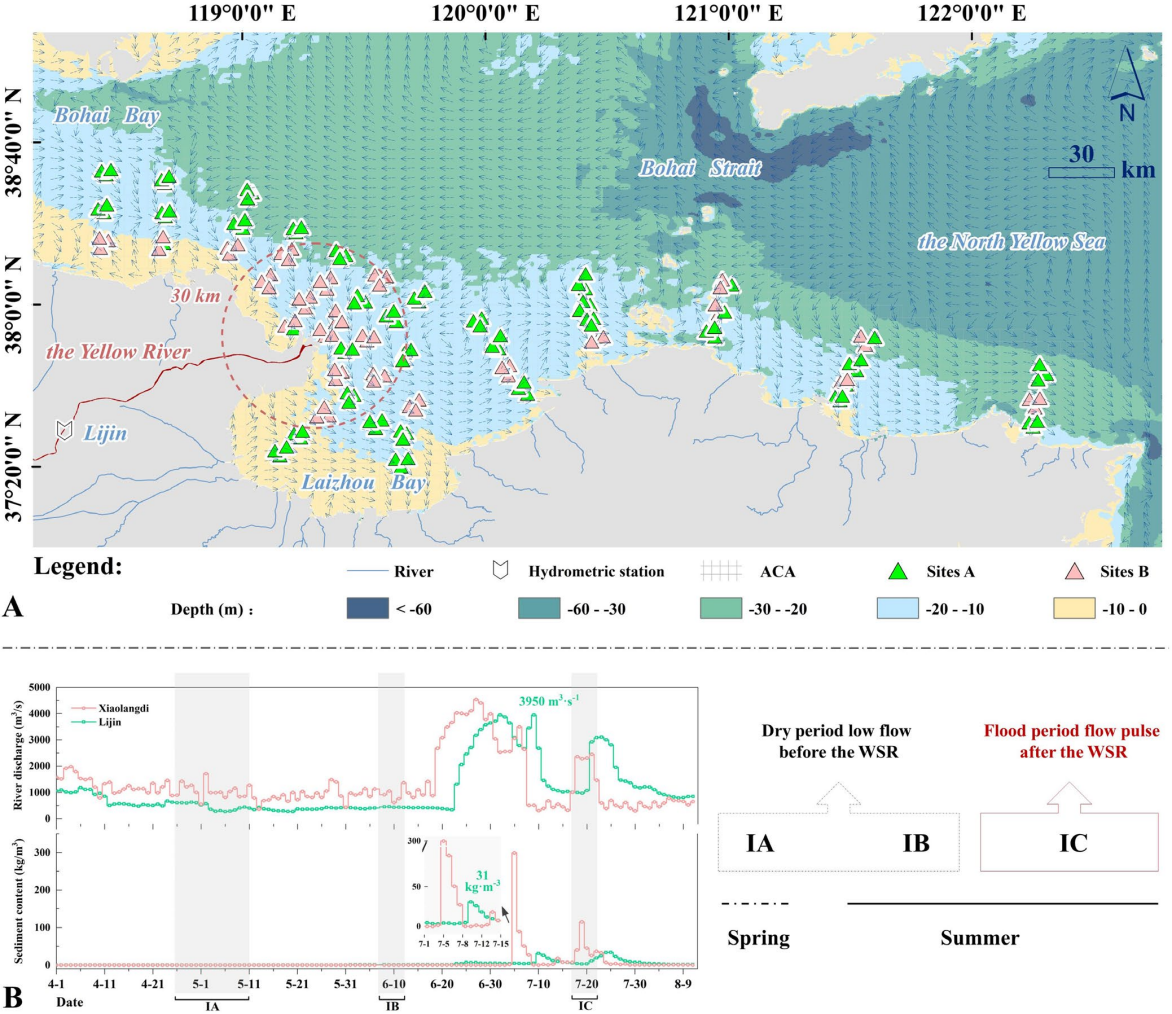
Sampling sites for the affected core area of the Yellow River flux (ACA). (A) The location of the ACA and sampling sites (Sites A: environmental investigation; Sites B: environmental and megabenthos investigation). (B) The daily freshwater discharge and sediment load data recorded at Xiaolangdi and Lijin hydrometric stations from the dry period to the artificial flood period in 2022 (left). The investigation design for this study (right). IA: the investigation cruise during the spring low‐flow period before the WSRS, IB: the investigation cruise during the summer low‐flow period before the WSRS, and IC: the investigation cruise after artificial flood period.

The survey was conducted in the affected core area of the Yellow River flux (ACA, 37°5′–38°45′ N, 118°10′–122°45′ E) (Figure 2A). The ACA is located in a typical temperate area, where the temperature of surface seawater can drop below 0°C in winter and increase to 28.5°C in summer, experiencing distinct seasons (https://mds.nmdis.org.cn/pages/home.html). The water depth in the study area ranges from 5 to 27 m (Figure 2A). The region is dominated by irregular diurnal tides, ranging from 1.5 to 3.0 m. Affected by the Lubei coastal current, the offshore flow rate is 8–20 cm s^−1^ (from west to east). A total of 26 rivers flow into this region, of which the Yellow River is the extreme proportion source of land‐based input. Under the influences of coastal current, the river flux of the Yellow River is transported from west to east along the southern of the Bohai Sea and can reach the Yellow Sea as far as possible in flood season (mainly occurring during the WSRS) (Wang et al. 2019; Liu, Qiao, et al. 2020; Liu, Yi, et al. 2020; Hou et al. 2024). Perennially, seawater exhibits characteristics such as significant seasonal temperature variations, low salinity, and high turbidity due to the combined effects of flux superimposition (Zhang et al. 2014; Li et al. 2021).

In 2022, the dry periods of the Yellow River mainly occurred from April 11 to June 18, and the mean values of river discharge and sediment load at the Lijin station were 440.96 m^3^ s^−1^ and 0.17 kg m^−3^, respectively (Figure 2B). Then the artificial flood was processed from June 19 to July 7, and the maximum runoff and sediment load were observed on July 2 and July 10, with flow rates reaching up to 3950 m^3^ s^−1^ and 31 kg m^−3^, respectively. An intense disturbance lasted for more than 20 days in the ACA during the flood period. The marine environment in the ACA is significantly influenced by the variation in Yellow River discharge into the sea during the dry periods (mainly occurring from mid‐April to mid‐June) and the artificial flood periods (after the discharge control of WSRS).

### 2.2 Sample Collection and Analysis

Three cruises that represent the dry periods (IA: the spring low‐flow period; IB: the summer low‐flow period) and the flood periods (IC: artificial flood by the WSRS) were conducted in the ACA on April 26–May 11, June 6–12, and July 18–22, 2022, respectively. All 159 stations were utilized for environmental investigation, and 56 stations were used for megabenthos investigation using the Agassiz trawl.

Megabenthos were gathered at selected sites using a bottom Agassiz trawl (2 m wide with a mesh size of 2 cm) (Figure 2B). The ship kept a straight course while bottom trawling (keeping speed ≤ 3 knots and for a continuous 30 min). The longitude and latitude of each trawling start and end point were tracked using GPS, and the trawling distance was calculated for each work. The biological samples were then rinsed, screened, and sorted in situ before being stored in the refrigerator at −20°C. In the lab, species identification work has been completed, and each species has been verified by three experts. Species abundance (ind.·km^−2^), biomass (wet weight, kg km^−2^), and other sample characteristics were recorded in detail. The number of severely damaged remains (with over 50% body loss, affecting biomass statistics) was recorded 69 individuals, 42 individuals, and 56 individuals during the IA, IB, and IC cruises, resulting in occurrence rates of only 0.67%, 0.19%, and 0.40%, respectively. Given this small proportion, the statistics for biomass of damaged specimens was supplemented by the mean of other undamaged individuals (Jac et al. 2021; Karlotta et al. 2023). It should be noted that the mollusks were weighed with shells, hermit crabs were weighed without shells, and the annelids were weighed without tubes. Before weighing, samples were placed on a chadless paper to absorb excess water and then weighed using electronic scales with a precision of 0.001 g. The above works were strictly carried out in accordance with the “Specification for Oceanographic Survey—Part4: Survey of Chemical Parameters in Seawater” (GB/T 12763.4‐2007).

The basic environmental parameters of bottom seawater temperature (Tem), salinity (Sa), pH, turbidity (Tu), depth, and chlorophyll *a* (Chl) were collected by the CTD profiler (911 plus, Sea‐bird Scientific, America) (Text B in Figure S1). Nutrient element concentrations, including seawater NO_3_^−^‐N, NO_2_^−^‐N, NH_4_^+^‐N, PO_4_^3−^‐P, and SiO_3_^2−^‐Si, were measured in the laboratory using an autoanalyzer (AutoAnalyzer III, Seal Analytical, Germany). Dissolved inorganic nitrogen (DIN) primarily consists of NO_3_^−^‐N, NH_4_^+^‐N, and NO_2_^−^‐N. N/P, Si/N, and Si/P ratios were calculated as the ratios of DIN to PO_4_^3−^‐P, SiO_3_^2−^‐Si to DIN, and SiO_3_^2−^‐Si to PO_4_^3−^‐P, respectively. Data of megabenthos and environmental parameters of all sampling sites have been kept on Figshare (https://figshare.com/s/6c98bd32b55d9e7dabf5). The above works were strictly carried out in accordance with the “Specification for Oceanographic Survey—Part6: Marine Biological Survey” (GB/T 12763.6‐2007).

### 2.3 Statistical Analysis

The data analysis was primarily implemented in the *R* language (IDE: *R*studio; version: 4.3.2). The biotic and environmental data were *log* (*x* + *1*) transformed to reduce the effect of extreme values. The differences between groups were first checked by the Kruskal–Wallis test, and then a Wilcoxon rank sum test was used as a post hoc analysis. The phylogenetic information was obtained at NCBI (https://www. ncbi.nlm.nih.gov/). The species phylogenetic tree was constructed using the package {phanorn} and then visualized using the iTOL software (https://itol.embl.de/). Co‐occurrence network analysis and visualization were conducted using Gephi software (version 0.10.1). The Mantel test between biodata and environmental parameters was analyzed in the package {linkET}. The basic data for the meta‐analysis of global marine benthos and dams in Figure 1 are from Mulligan, van Soesbergen, and Sáenz (2020) (Appendix S1), Stratmann et al. (2020) (Appendix S2), and Wang et al. (2022) (Appendix S3). After data screening, the biological abundance (ind.·m^−2^) and biomass (wet weight, g·m^−2^) data of 45,182 and 6578 marine benthos samples were, respectively, selected for statistics. Other cartographic works were mainly plotted by ArcGIS 10.8 and Origin 9.1 software.

To reveal the community characteristics of megabenthos in the study area, 24 indices were adopted (Table S1). Of them, eight community alpha diversity indices were used to characterize the biodiversity of the megabenthos community, including species richness (*S*), species abundance (*Sa*), species biomass (*Sb*), Shannon–Wiener diversity index (*H´*), Margalef species richness index (*d*), Pielou species evenness index (*J´*), Simpson diversity index (*D*), and the inverse of Simpson index (*D´*). Other eight indices were used to characterize the phylogenetic diversity of the megabenthos community, including phylogenetic diversity (*PD*), mean pairwise distance (*MPD*), mean nearest taxon distance (*MNTD*), nearest relative index, phylogenetic species variability (*NRI*), phylogenetic species evenness (*PSV*), phylogenetic species richness (*PSR*), and phylogenetic species clustering (*PSC*). Another eight indices were used to characterize the stability of the megabenthos community, including the community stability index (*ICV*), positive cohesion (*C_pos*), negative cohesion (*C_neg*), total cohesion (*C_total*), average variation degree (*AVD*), robustness for random species removal (*Robustness_R*), robustness for dominant species removal (*Robustness_Y*), and vulnerability metrics (*Vulnerability*).

In addition, three comprehensive indices of communities at the levels of biodiversity, phylogeny, and stability were set and measured in this study, which were *C‐diversity*, *C‐phyl*, and *C‐stability*, respectively. The indices of *C‐diversity*, *C‐phyl*, and *C‐stability* were calculated according to the entropy weight method based on the above 24 indices (Table S1; Text A in Figure S1).

## 3 Results

### 3.1 Megabenthos Composition

**FIGURE 3:**
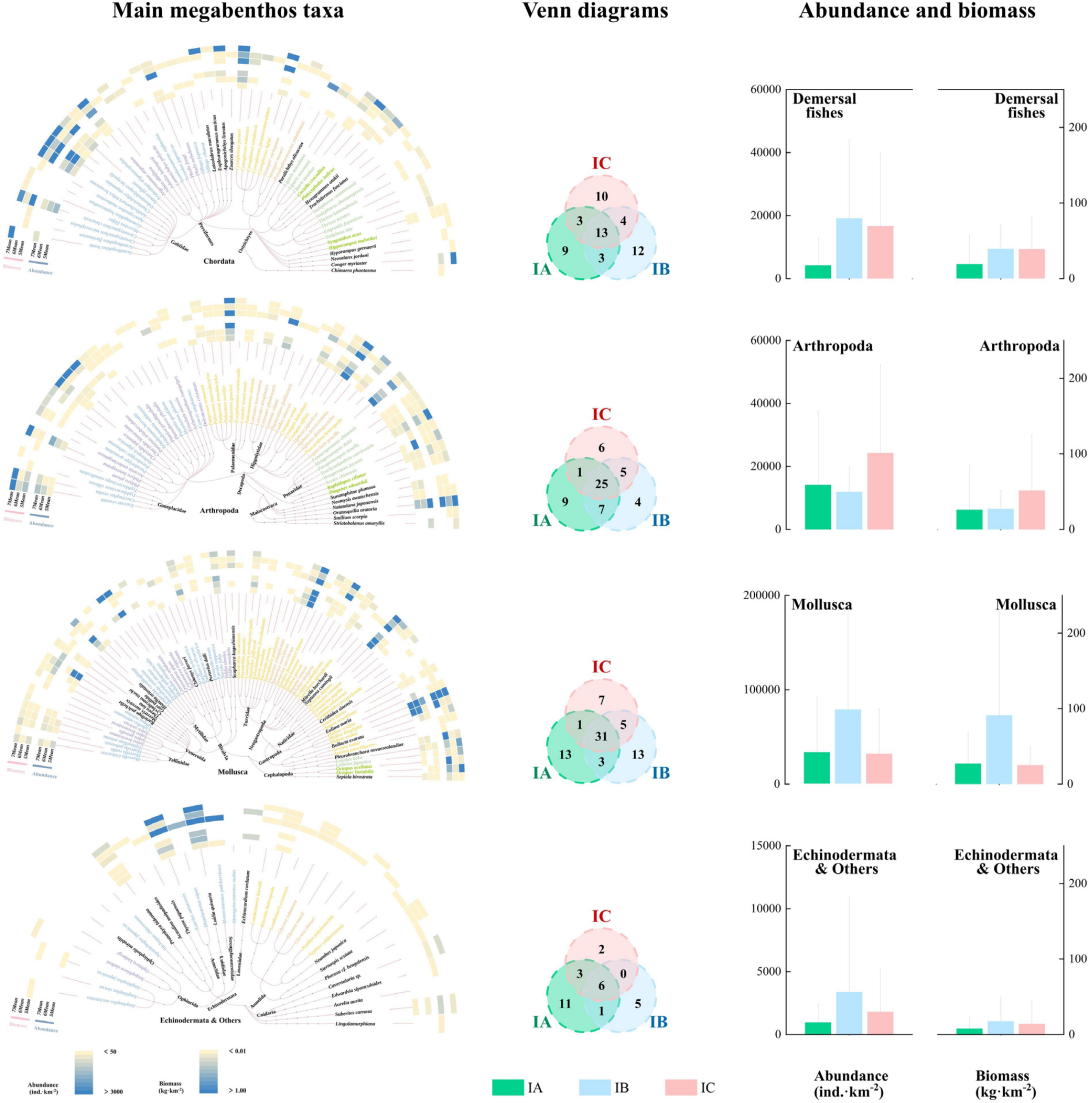
The composition, abundance, and biomass of megabenthos community during the periods of IA, IB, and IC in the ACA. Left region (main megabenthos taxa): Changes in species abundance and biomass of the major microbenthic taxa, which from top to bottom are Chordata, Arthropoda, Mollusca, Echinodermata, and others, respectively. Middle region (Venn diagrams): Species Venn diagrams among IA, IB, and IC of each major microbenthic taxa. Right (abundance and biomass): Comparison of megabenthos abundance and biomass among IA, IB, and IC of each major microbenthic taxa.

In ACA, a total of 10,262, 21,952, and 13,982 megabenthos were collected during the periods of IA, IB, and IC, respectively (Figure 3). Samples were identified as 139 species (belonging to 15 classes and 46 orders), 137 species (belonging to 11 classes and 40 orders), and 122 species (belonging to 12 classes and 41 orders) of IA, IB, and IC, respectively. The average abundance during IA, IB, and IC was 53,023, 113,425, and 76,257 ind.·km^−2^, respectively. The average biomass during IA, IB, and IC was 80.20, 175.32, and 130.20 kg·km^−2^, respectively. A maximum average abundance and biomass values both were observed during the IB period. A total of 13 demersal fishes (*Lateolabrax*, *Argyrosomus*, and others), 25 arthropods (*Charybdis*, *Portunus*, and others), 31 mollusks (*Neverita*, *Octopus*, and others), and other six species (mainly including *Luidia* and *Asterias*) were observed during all three periods. The main taxonomic group changed over different periods, especially demersal fishes and mollusks.

**FIGURE 4:**
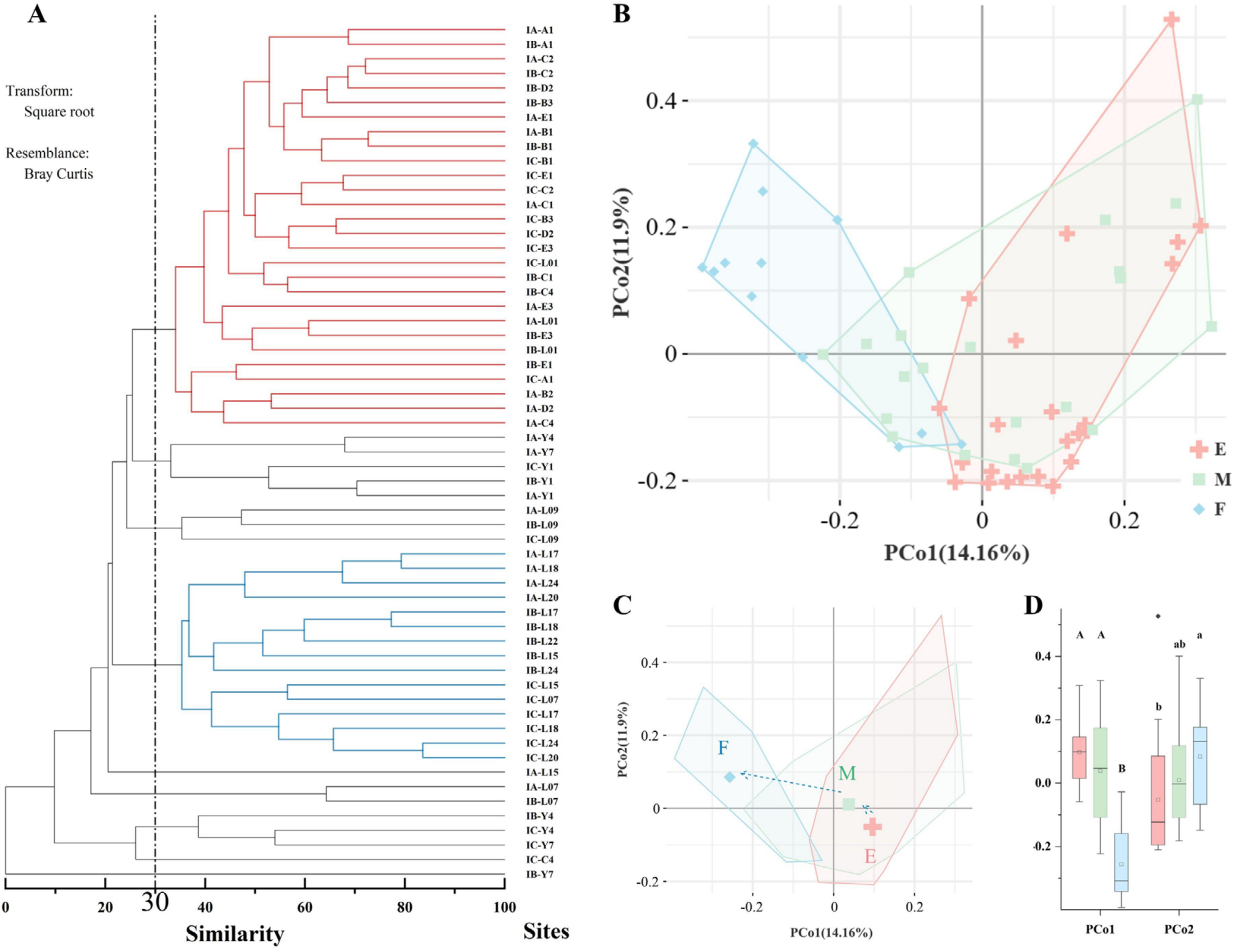
Megabenthic community structure and variation across the estuarine gradient in the ACA. (A) Using pooled abundance data, a cluster analysis was performed on the megabenthos community within the ACA. (B) The principal coordinate analysis (PCoA) of the megabenthos communities was based on different regions: the estuary region (E), the transition region (M), and the region away from the estuary (F). (C) The centroid location of PCoA at E, M, and F regions. (D) A permutational multivariate ANOVA test is conducted to show the variation in megabenthos assemblages among different regions for the first axis (PCo1) and the second axis (PCo_2_), respectively.

The hierarchical cluster analysis (Figure 4A) and the principal coordinate analysis (PCoA) (Figure 4B) results showed a spatial difference in the community structure of megabenthos. According to community similarity and regional differences, the megabenthos in the ACA could be divided into three main communities: the estuarine community (E), the nonestuarine community (F), and the transitional community (M) at a similarity level of more than 30%. The centroid location of PCoA showed a significant shift between E and F (Figure 4C). Significant differences were observed in both the first axis (PCo1) and the second axis (PCo2) between the communities of E and F by the permutational multivariate ANOVA test (*p* < 0.05) (Figure 4D).

### 3.2 Biodiversity, Phylogenetic Diversity, and Stability

To investigate the geospatial characteristics of megabenthos fauna in the estuary, the biodiversity, phylogeny, structure, and stability of communities were analyzed based on the results of PCoA and cluster analysis. Entropy weighting showed that the *S*_*b*_ (*w* = 0.29) and the *S*_*a*_ (*w* = 0.27) possess the highest weights among the eight alpha indices (Table S2). *C‐diversity* exhibited a high standard deviation in IA, IB, and IC, indicating a high distribution variation in biodiversity within the ACA. The *C‐diversity* fluctuated sharply (from 0.04 to 0.62) in the estuarine community (mainly within 30 km of the estuary), whereas the changes in the nonestuarine community (especially 80 km outside the estuary) were relatively stable (ranging from 0.22 to 0.41) (Figure S1). Specifically, the *C‐diversity* values of the estuarine community in the IB period (IB‐E) and IC period (IC‐E) were significantly higher than those of the IA period (IA‐E) (*p* < 0.05) (Figure S1). In addition, a significant difference was observed between the northern side estuary (IC‐EN) and the southern side estuary in the IC (IC‐ES) (*p* < 0.05). This internal difference was not observed in either IA‐E or IB‐E.

The results of entropy weighting showed that the *PSR* (*w* = 0.20) and the *PSE* (*w* = 0.19) possess the highest weights among the phylogenetic diversity indices (Table S3). The *C‐phyl* values of the megabenthic community in IB (0.68 ± 0.12) and IC (0.64 ± 0.16) were higher than those of IA (0.56 ± 0.13) in the entire region. The comparison of estuarine communities in different periods showed significant differences in *PSE*, *PSV*, and *MNTD* between IA‐E and IB‐E (*p* < 0.05) (Figure S2). In addition, the significant differences in *PD*, *PSR*, *PSE*, *PSV*, and *MNTD* were observed between IC‐EN and IC‐ES (*p* < 0.05).

The *C_neg* index possessed the highest entropy weighting (*w* = 0.28) among the indices of community stability (Table S4). The *C‐stability* value of IB (0.56) was higher than that of IC (0.46) and IA (0.41). The comparison of estuarine communities showed that the *AVD*, *ICV*, and *C_pos* of IB‐E and IC‐EN were higher than those of IA‐E and IC‐ES (Figure S3). Significant differences were observed in *ICV*, *C_pos*, *Robustness_R*, and *Robustness_Y* between IC‐EN and IC‐ES (*p* < 0.05), which was consistent with the results of alpha diversity and phylogenetic diversity on the whole.

**FIGURE 5:**
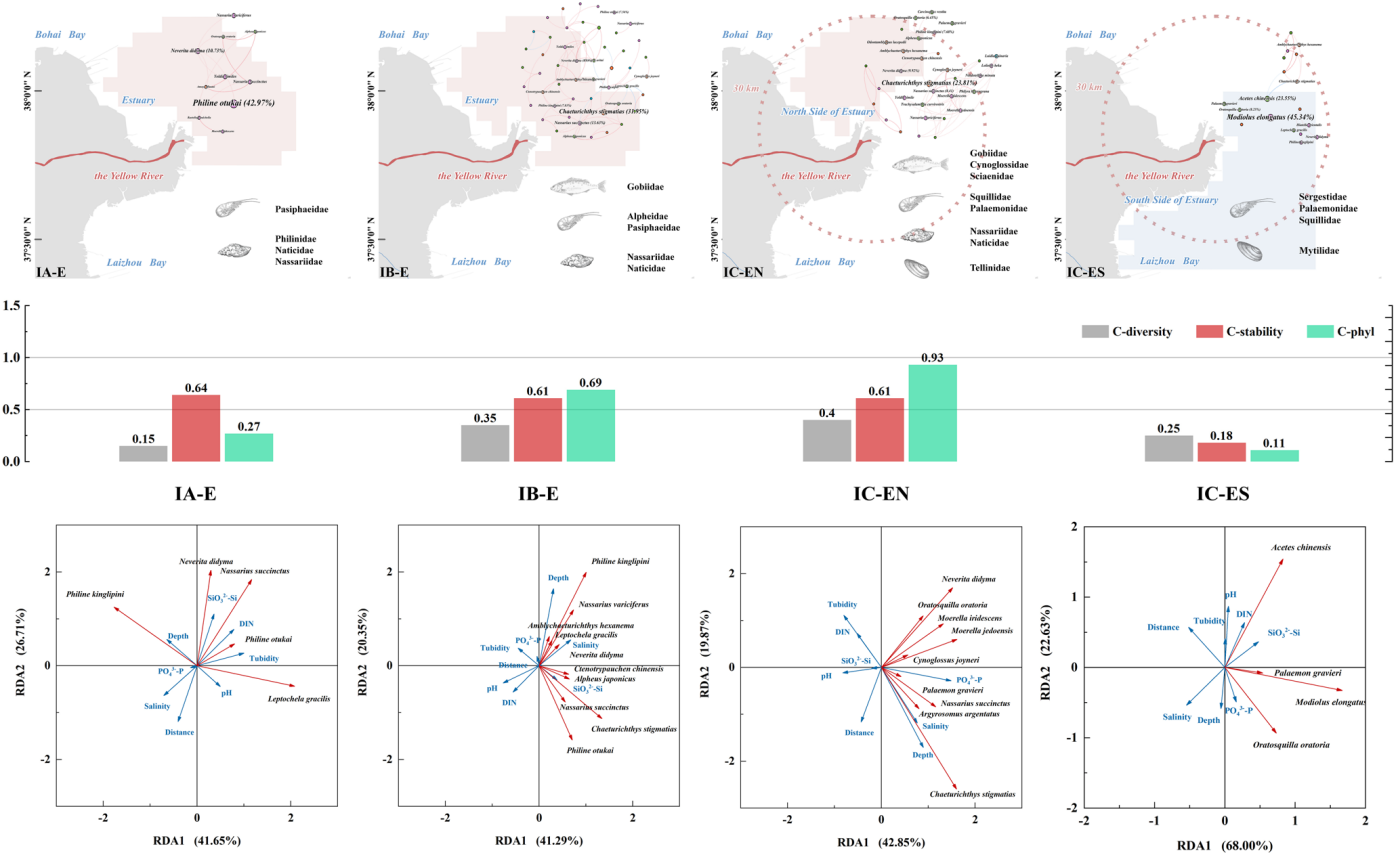
Community structure and characteristics of megabenthos in the Yellow River Estuary at different periods (IA, IB, IC‐EN, and IC‐ES). Four diagrams in the top row: Community structure and dominant taxa of megabenthos in the estuary at different periods. Bar charts in the middle raw: The comprehensive indices for biodiversity (*C‐diversity*), phylogenetic diversity (*C‐phyl*), and stability (*C‐stability*) of megabenthos community. Four diagrams in the bottom row: Redundancy analysis (RDA) correlation plots of the main dominant species (red line) with environmental variables (blue line) in the estuarine region at different periods.

As a whole, the *C‐diversity* values of the estuarine community during different periods followed IC‐EN (0.40) > IB‐E (0.35) > IC‐ES (0.25) > IA‐E (0.15) (Figure 5). Similarly, the *C‐phyl* values followed IC‐EN (0.93) > IB‐E (0.69) > IA‐E (0.27) > IC‐ES (0.11). The *C‐stability* values followed IA‐E (0.64) > IC‐EN and IB‐E (0.61) > IC‐ES (0.18). These changes in IB‐E, IC‐EN, and IC‐ES were consistent; the former two communities showed a high level of biodiversity, phylogenetic diversity, and stability, whereas the indices in IC‐ES exhibited a sharp contrast. Unique to the indices in IA‐E was a low value of *C‐diversity* and *C‐phyl* against a high *C‐stability* value.

### 3.3 Analysis of Factors Affecting the Megabenthic Community

For benthic environmental conditions among different periods, there were significant differences in temperature, pH, chlorophyll *a*, PO_4_^3−^‐P, NO_3_^−^‐N, and DIN between periods IA and IB (*p* < 0.05) (Table S5). Significant differences in seawater turbidity, chlorophyll *a*, NH_4_^+^‐N, NO_3_^−^‐N, PO_4_^3−^‐P, SiO_3_^2−^‐Si, DSi/DIN, and DSi/DIP were observed between periods IB and IC (*p* < 0.05). In terms of spatial distribution characteristics, the range of partial seawater parameters (especially salinity, turbidity, and nutrient composition) during the IC period underwent significant changes compared to the overall fluctuation of seawater parameters during the IB period.

The Mantel's test was conducted among major taxa of megabenthos at the family level, correlating with environmental parameters using pooled abundance data in the ACA (Figure S4). The results showed that, on the one hand, there was collinearity and significant correlations between some environmental parameters. According to the correlation between the “Distance” and other parameters, a significant gradient correlation with the distance from the estuary existed in water depth (*p* < 0.01), salinity (*p* < 0.01), turbidity (*p* < 0.05), silicate content (*p* < 0.01), nitrogen nutrient content (*p* < 0.01), and the nutrient structure of N/P/Si (*p* < 0.01) in the ACA. On the other hand, some taxa (i.e., Apogonidae, Alpheidae, Sergestidae, Penaeidae, Naticidae, Mytilidae, and others) showed a significant correlation with the distance from the estuary, salinity, turbidity, and nutrient structure (*p* < 0.05).

According to the network analysis for the co‐occurrence of megabenthos in the estuarine community, the average degree (d), average path length (l), clustering coefficient (c), and modularity index (m) of the network in IA‐E was 14.49, 1.78, 0.45, and 0.27, respectively (Figure S5IA‐E; Text B in Figure S1). There were five dominant species, including *Leptochela gracilis* (*Y* = 0.18), *Philine otukai* (*Y* = 0.17, abundance ratio = 42.97%), and others (Table S6), belonging to four families (Figure 5IA‐E). In IB‐E, the d, l, c, and m were 4.79, 3.17, 0.26, and 0.60, respectively (Figure S5IB‐E). There were 10 dominant species, including *Chaeturichthys* *stigmatias* (*Y* = 0.28, abundance ratio = 31.95%), *Nassarius succinctus* (*Y* = 0.19), *Neverita didyma* (*Y* = 0.11), and others belonging to five families (Figure 5IB‐E). In IC‐EN, the d, l, c, and m were 9.67, 2.46, 0.47, and 0.34, respectively (Figure S5IC‐EN). The counterparts were 11.83, 2.12, 0.33, and 0.34 in IC‐ES, respectively (Figure S5IC‐ES). There were nine dominant species in IC‐EN: *C. stigmatias* (*Y* = 0.17), *N. succinctus* (*Y* = 0.08), and others belonging to eight families (Figure 5IC‐EN). However, only four dominant species existed in IC‐ES: *Modiolus elongatus* (*Y* = 0.36, abundance ratio = 45.34%), *Acetes chinensis* (*Y* = 0.19), *Oratosquilla oratoria* (*Y* = 0.07), and *Palaemon graviera* (*Y* = 0.04) belonging to four families (Figure 5IC‐ES).

Redundancy analysis (RDA) was used to investigate relationships between the dominant species (*Y* ≥ 0.02) and key environmental parameters in each period (Table S6). The first two axes in IA‐E explained 41.65% and 26.71% of the variation, respectively (Figure 5IA‐E). The first axis was mainly formed by turbidity, whereas the second axis was influenced primarily by the distance from the estuary, silicate, and salinity. Most dominant species were associated with a close estuary distance, low salinity, and high silicate environment. The first two axes in IB‐E explained 41.29% and 20.35% of the variation (Figure 5IB‐E). The first axis was mainly contributed by salinity, whereas the second was primarily contributed by water depth and DIN. Most dominant species were associated with high silicate, high salinity, and low turbidity. *C. stigmatias* , which was the absolute dominant species in the IB period (abundance ratio = 31.95%, *Y* = 0.28), was positively correlated with high silicate and low turbidity. In IC‐EN, the most dominant species were associated with high salinity, high phosphate, and low turbidity, which was consistent with IB‐E (Figure 5IC‐EN). The communities of IB‐E and IC‐EN were consistent in that most of the dominant species are associated with the benthic condition of low salinity and high silicate.

## 4 Discussion

### 4.1 Key Areas for Marine Megabenthos Impacted by River Discharge: Estuaries

Due to the interface of marine, terrestrial, and large river, along with the river regulation, the estuary has emerged as an ecologically sensitive region (Zhang et al. 2021; Li et al. 2023). Our study revealed that a variation in both benthic conditions and megabenthic fauna existed in the Yellow River Estuary. A variety of environmental parameters (such as seawater salinity, turbidity, nutrient contents, and stoichiometric ratios) showed obvious gradient changes with the distance from the estuary (Figure S4). Furthermore, based on the results of hierarchical cluster analysis and PCoA analysis, the megabenthos were classified into three major faunas in the ACA (Figure 4), which were the estuarine community, the nonestuarine community, and the transitional community, with a similarity level above 30%. Combining the spatial distribution of estuarine community positions and the variation of the *C‐diversity* index at the distance from the estuary, it was considered that within 30 km from the estuary is the main distribution region of the estuarine community (Figures S1 and 5). This distance was consistent with studies on diluted water and fronts in this region (Wang et al. 2017; Liu, Qiao, et al. 2020; Liu, Yi, et al. 2020; Chang et al. 2022).

On the one hand, the species composition of the estuarine community differs significantly from that of the nonestuarine community. The *C‐diversity* fluctuated sharply (from 0.04 to 0.62) in the estuarine community, whereas the changes in *C‐diversity* in the nonestuarine community were relatively stable (ranging from 0.22 to 0.41) (Figure S1). Estuaries, as unique zones where freshwater and seawater converge, normally exhibit dynamic and complex physical and chemical conditions, such as salinity, sediment type, and water flow velocity (Deng et al. 2017; Wang et al. 2017). These specific environmental conditions can shape distinctive biological communities that possess a high degree of variability and adaptability to changing environments (Wang et al. 2021; Zhang et al. 2021). In contrast, nonestuarine environments generally present more stable conditions, where the composition of biological communities exhibits different patterns in species adaptability and ecological niche occupation (Martins et al. 2022; De Lima et al. 2024).

On the other hand, the biodiversity of megabenthos in the estuary exhibits a significant difference among different periods (Figure S1). Studies have shown that during periods of huge river discharge, an increased sediment load and nutrient influx can enhance the food availability for marine benthos, thereby potentially increasing biodiversity (Baptista et al. 2010). Conversely, during low discharge periods, reduced sediment and nutrient inputs may lead to a decrease in benthic diversity, especially in the spring (Scharler, Bownes, and Jerling 2023). The high variability in estuarine biodiversity highlights the sensitivity of megabenthic fauna to river discharge.

### 4.2 Dynamics and Variability of Megabenthos Fauna in the Yellow River Estuary

During the IA period, the *C‐diversity* and *C‐phyl* indices of megabenthos communities in the estuary reached relatively low levels (Figure 5). The estuarine community manifested by few species, low biomass, and a large mean phylogenetic distance. Concretely, the dominant taxa were Pasiphaeidae, Philinidae, Naticidae, and Nassariidae during this period (Table S6). Among them, *P. otukai* and *Philine kinglipini* account for 42.97% and 10.84% of the abundance, making them the absolute dominant species. Spring is a critical period for the growth and development of Philinidae. The input of terrestrial organic debris from the Yellow River and tidal creeks (Wang, Lv, and Li 2018), along with the suitable soft‐bottom environment in the estuarine area, serves as an important food source and environmental foundation for their explosive growth (Li et al. 2017; Duan et al. 2024).

However, in contrast to low *C‐diversity* and *C‐phyl*, estuarine communities during this period exhibited high *C‐stability* values (Figure 5). Combining co‐occurrence network analysis revealed that although species richness is low, the close interspecific connections lead to a high stable community (Figure S5; Text B in Figure S1). RDA analysis showed that the most dominant species are associated with low salinity, high silicate, and high DIN (Figure 5). The constituent species of estuarine community hail from various evolutionary lineages, each having evolved distinct physiological and ecological adaptations to navigate the dynamic and harsh conditions typical of estuarine ecosystems (Rodrigues et al. 2022; Wang et al. 2023). The extensive adaptive divergence is necessitated by the fluctuating conditions characteristic of estuary (Raimonet and Cloern 2017). In the spring low‐flow period, the estuarine community was dominated by small shrimps and mollusks, which adapted to the habitat near the estuary with relatively low salinity and high nitrogen and silicate nutrient levels (Figure 5IA‐E).

Different from spring, the *C‐diversity* and *C‐phyl* values were both relatively high during the IB period (Figure 5IB‐E). The dominant taxa of communities during this period included demersal fishes (i.e., Gobiidae), shrimps (i.e., Alpheidae and Pasiphaeidae), and mollusks (i.e., Nassariidae and Naticidae). Among them, *C. stigmatias* accounts for 31.95% of the abundance, making Gobiidae the absolute dominant taxa. On the one hand, individual morphological statistics indicate that the large individuals represent 11.39% of Gobiidae, whereas the small individuals account for 88.61%. The estuarine area from June to September is an important breeding ground for bottom fishes such as *C. stigmatias* , and the proportion of small individuals is very high. On the other hand, the stomach content observation of *C. stigmatias* showed that the stomach contents of large individuals mainly consist of *Alpheus japonicus* and *Palaemon gravieri* , whereas those of small individuals primarily include *Yoldia similes* , *Glauconome primeana* , *Latreutes planirostris* , and some polychaetes. The increase in temperature and sustained nutrient input can facilitate algal blooms that enhance primary productivity during this period (Wu et al. 2016; Zhang et al. 2021). This elevation in productivity provides a foundation for increased species richness and biomass in the estuary (Yi, Zhao, et al. 2023). Furthermore, new ecological niches become available or more pronounced (Mbandzi et al. 2018). This expansion in ecological niches allows for more species coexistence and diversification, thereby augmenting phylogenetic diversity.

Similarly, co‐occurrence network analysis showed a closely interspecific association, and the community had relatively high stability (Figure S5; Text B in Figure S1). Additionally, most fish and invertebrates have adapted to spawn from spring to early summer, capitalizing on the increased availability of food and suitable nursery habitats for their offspring (Raimonet and Cloern 2017; Cariou et al. 2020), especially Gobiidae in the study area. Estuarine communities during this period are characterized by a high level of species richness, abundance, biomass, biodiversity, and a relatively matured phylogeny. The estuarine megabenthic fauna is dominated by small fishes, shrimps, and mollusks, which adapted to the estuarine conditions with relatively high salinity and silicate nutrient levels (Figure 5).

Furthermore, the values of *C‐diversity*, *C‐phyl*, and *C‐stability* showed significant spatial differences between the northern and southern sides of the estuary during the IC period (Figure 5IC‐EN,IC‐ES). Compared to the northern community, the mean values of *C‐diversity*, *C‐phyl*, and *C‐stability* in the southern decreased by 37.50%, 70.49%, and 88.17%, respectively. Under the influence of flux pulse and the nearshore current (Lubei coastal current), a channel of flux entering the sea from north to south is formed on the south side of the estuary (basically located in the nearshore area within 15 m of the isobath) (Zhang et al. 2014; Li et al. 2021). Flux into the sea during the flood season dramatically changes the benthic habitat and fauna from southern sides of the estuary to the Laizhou Bay (Text C in Figure S1) (Hou et al. 2024; Sun et al. 2024). Therefore, differences were observed in the biodiversity, phylogeny, and homeostasis between the two sides. The main megabenthic taxa in the northern were mainly the medium and large benthic fishes and crustaceans (i.e., Gobiidae, Cynoglossidae, and Palaemonidae). On the one hand, the community characteristics of IC‐EN were similar to those of IB‐E. Meanwhile, the northern community had higher biodiversity and phylogenetic diversity compared to IB‐E. In contrast, IC‐ES was mainly composed of Sergestidae ( *A. chinensis* ), Palaemonidae ( *P. gravieri* ), and Mytilidae (*M. elongatus*) (Table S6). It was consistent with the study of food web structure and energy flow by Liu, Qiao, et al. (2020); Liu, Yi, et al. (2020); and Yi et al. (2021) between two sides. All topological indices of the food web in the northern were higher than those in the southern after the flood period. The input and diffusion of sediment and nutrients carried by flux pulse led to a decrease in trophic levels of major consumers and changes in the structure of estuarine organisms (Liu, Qiao, et al. 2020; Liu, Yi, et al. 2020).

The co‐occurrence network analysis showed that the number of key nodes, clustering coefficient, and modularity index of the network in the northern community are higher than those in the southern (Figure S5; Text B in Figure S1). RDA analysis showed that the most dominant species in the northern were associated with low pH and high phosphate concentration (Figure 5). The northern fauna was well maintained in homeostasis, composed of fishes, shrimp, and crustaceans that adapted to the relatively high phosphate and low silicate environment. In the estuary, the southern fauna had a very low species richness and biodiversity, associated with a low salinity and high silicate concentration environment. Artificial flooding–induced changes in benthic habitats led to significant impacts on the estuarine megabenthos fauna during the flood period.

### 4.3 Impact of Artificial Flood on Estuarine Ecosystems in the Yellow River Estuary

From the perspective of community geography, the typical estuarine fauna was mainly distributed within 30 km of the Yellow River Estuary during the spring and summer low‐flow periods. After the artificial flood in summer, the spatial pattern of the estuarine fauna changed due to the influence of the flux pulse. The estuarine community maintained a good steady state within 30 km on the northern side (Figure 5IC‐EN). The stability of the estuarine community on the southern side was damaged seriously by artificial flood (WSRS) (Figure 5IC‐ES). The discharge characteristics of diluted water and nutrients from the Yellow River in coordination with the nearshore current reshaped the distribution pattern of marine megabenthic fauna in the estuary.

Our study showed that the sudden increases in freshwater and sediment discharge over a short term during artificial floods after chronic low flow were not conducive to estuarine benthic ecology. The Yellow River Estuary serves as a crucial biological habitat, spawning ground, and nursery area for the Bohai Sea, exhibiting extremely high biodiversity during the summer period (Zhang et al. 2021; Wu et al. 2024). The current water and sediment regulation process, characterized by its short duration of artificial flooding (mainly within 20 days) and abrupt changes in discharge, has a significant impact on this ecologically critical area (Han et al. 2024; Qiao et al. 2024), particularly on the southern side of the estuary. Research conducted by Ren et al., Liu et al., Yi et al., Zhang et al., Yang et al., Hu et al., and Li et al. on phytoplankton, benthic organisms, fish, ichthyoplankton, energy flow, and fishery resources, respectively, in the region has emphasized this issue from various perspectives (Ren et al. 2016; Liu, Qiao, et al. 2020; Liu, Yi, et al. 2020; Yi et al. 2021; Yi, Gao, et al. 2023; Yi, Zhao, et al. 2023; Zhang et al. 2021; Yang et al. 2023; Hu et al. 2024; Li et al. 2024). Considering that the current water and sediment regulation projects have sufficiently fulfilled their function of sediment clearing downstream (Cheng et al. 2022; Han et al. 2024), the discharge strategy should be adjusted by ecological considerations across the entire region of the river basin. Implementing phased or pulse water releases to more closely mimic the natural seasonal flow patterns of rivers could be beneficial. Reducing the rate and total volume of water released during the artificial flood season, or avoiding large‐scale discharges during ecologically sensitive periods, would mitigate the abrupt impacts on the estuarine ecosystem.

## 5 Conclusion

Our study examined the impacts of river discharge and artificial flooding on the megabenthic fauna in the Yellow River Estuary through three comprehensive indices synthesized based on 24 basic indices. The estuarine megabenthic community showed variations in biodiversity, phylogenetic diversity, and community stability across three discharge periods.

During the spring low‐flow period, the estuarine community was characterized by low biodiversity but high stability, followed by an increased biodiversity and phylogenetic diversity exhibited in the summer. After the flood period, the community was bifurcated into distinct northern and southern groups. The northern community maintained high biodiversity and community homeostasis, whereas the stability of the southern estuarine community was seriously damaged.

The typical estuarine communities were mainly distributed in the key area within 30 km of the Yellow River Estuary during the low‐flow periods. During the flood period, the affected area of river discharge extended over 30 km along the southern side of the estuary. The megabenthic fauna in the Yellow River Estuary was strongly affected by artificial flood regulation, highlighting the dramatic changes of the bottom seawater. A more ecologically considerate discharge management of the Yellow River, which takes into account the estuary ecology, is imperative.

## Author Contributions

**Debin Sun:** conceptualization (lead), data curation (lead), formal analysis (lead), investigation (lead), methodology (lead), software (lead), visualization (lead), writing – original draft (lead). **Qinglu Fu:** formal analysis (supporting), investigation (supporting), methodology (supporting). **Jiao Wang:** formal analysis (supporting), supervision (lead). **Linlin Chen:** formal analysis (equal), funding acquisition (lead), supervision (equal). **Jing Chen:** formal analysis (equal), supervision (equal). **Yilin Wang:** formal analysis (supporting), investigation (supporting). **Baoquan Li:** conceptualization (lead), formal analysis (lead), funding acquisition (lead), methodology (lead), project administration (lead), resources (lead), writing – original draft (lead), writing – review and editing (lead).

## Conflicts of Interest

The authors declare no conflicts of interest.

## Supporting information


**Appendix S1.** https://doi.org/10.6084/m9.figshare.c.4648214.v1.

**Appendix S2.** https://doi.org/10.5061/dryad.gb5mkkwm6.

**Appendix S3.** https://doi.org/10.5281/zenodo.6163413.

**Figure S1.** The data statistics of C‐diversity the ACA for three cruises (IA, IB, and IC). (A) Variation of the C‐diversity index at a distance from the estuary. B: Comparison of C‐diversity index in different IA, B, C periods. (C) Comparison of C‐diversity in the estuary region (E), the transition region (M), and the region staying away from the estuary (F). (D) C‐diversity of estuary region in different periods. * denotes a significant difference between the two regions (*p* < 0.05).
**Figure S2.** The leading phylogenetic diversity indices of the megabenthos community. PD, PSR, PSE, PSV, MPD, and MNTD denote the phylogenetic diversity, phylogenetic species richness, phylogenetic species evenness, phylogenetic species variability, mean pairwise distance, and mean nearest taxon distance, respectively. *denotes a significant difference between the two regions (*p* < 0.05).
**Figure S3.** The stability indices of megabenthic community. AVD, ICV, C_pos, Robustness_R, Robustness_Y, and Vulnerability denote the average variation degree, community stability index, community positive cohesion, network robustness (the proportion of species remaining after 50% of the species are randomly removed from each community), network robustness (the proportion of species remaining after the dominant species are removed from each community), and community vulnerability. *denotes a significant difference between the two regions (*p* < 0.05).
**Figure S4.** Pearson’s analysis of environmental parameters (left) and Mantel’s test among dominant taxa of megabenthos with environmental parameters (right) based on pooled abundance data. Note: Major taxa of megabenthos denote major family groups with more than 10% of the species occurrence frequency in pooled abundance data.
**Figure S5.** Network analysis for the co‐occurrence of megabenthos in the estuarine community in each period (IA, IB, and IC) based on the relative abundance. The (d, l, c, m) denote the average degree, average path length, clustering coefficient, and modularity index of the network, respectively.
**Table S1.** Overview of different indices of megabenthos community employed in this study.
**Table S2.** The weight and mean values (mean ± SD) of the comprehensive biodiversity index (C‐diversity) for three cruises (IA, IB, and IC).
**Table S3.** The weight and mean values (mean ± SD) of the comprehensive index of phylogenetic diversity (C‐phyl) for three cruises (IA, IB, and IC).
**Table S4.** The weight and mean values (mean ± SD) of the comprehensive index of stability (C‐stability) for three cruises (IA, IB, and IC).
**Table S5.** Ranges, mean values (±SD), and variation coefficient values (CV) of environmental parameters in the bottom seawater layers during IA, IB, and IC periods.
**Table S6.** The dominant species and dominance degree (Y) of the estuarine community in each period (IA, IB, and IC).
Texts (A–C): (A) The calculation statements of each index in Table S1. (B) The statements of network analysis for co‐occurrence in Figures 5 and S5. (C) The statements of seawater environmental parameters in Table S5.

## Data Availability

All associated raw data to reproduce the results presented in this article are available on Figshare (https://figshare.com/s/6c98bd32b55d9e7dabf5).
