# Supplementary material for: How Marine Megabenthos Fauna Responds to River Discharge and Artificial Flood in Large River Estuary
Source: Ecol Evol. 2025 Jan 8;15(1):e70755. doi: 10.1002/ece3.70755 (PMC11707399; doi:10.1002/ece3.70755)
Supplement: Supplementary file 3 — Appendix S3. https://doi.org/10.5281/zenodo.6163413. [file ECE3-15-e70755-s001.zip › GeoDAR_v10_v11/Readme_GeoDAR_v10_v11.pdf]

GeoDAR: Georeferenced global Dams And Reservoirs dataset for bridging attributes and geolocations

Versions 1.0 and 1.1 (https://doi.org/10.5281/zenodo.6163413). April 13, 2022

Authors: Jida Wang[1], Blake A. Walter[1], Fangfang Yao[2], Chunqiao Song[3], Meng Ding[1], Abu Sayeed Maroof[1], Jingying Zhu[3], Chenyu Fan[3], Jordan M. McAlister[4], Md Safat Sikder[1], Yongwei Sheng[5], George H. Allen[6], Jean-François Crétaux[7], and Yoshihide Wada[8]

[1]Department of Geography and Geospatial Sciences, Kansas State University, Manhattan, Kansas, USA

[2]Cooperative Institute for Research in Environmental Sciences (CIRES), University of Colorado Boulder, Boulder, Colorado

[3]Nanjing Institute of Geography and Limnology, Chinese Academy of Sciences, Nanjing, China

[4]Department of Geography, Oklahoma State University, Stillwater, Oklahoma, USA

[5]Department of Geography, University of California, Los Angeles (UCLA), Los Angeles, California, USA

[6]Department of Geography, Texas A&M University, College Station, Texas, USA

[7]Laboratoire d'Études en Géophysique et Océanographie Spatiales (LEGOS), Centre National d'Études Spatiales (CNES), Toulouse, France

[8]International Institute for Applied Systems Analysis (IIASA), Laxenburg, Austria

Correspondence to: Jida Wang (jidawang@ksu.edu)

Data components

Folder "GeoDAR_v10_v11" contains two consecutive, peer-reviewed versions (v1.0 and v1.1) of the Georeferenced global Dams and Reservoirs (GeoDAR) dataset:

- **GeoDAR_v10_dams** (in both shapefile format and the comma-separated values (csv) format): GeoDAR version 1.0, including 22,560 dam points georeferenced based on the World Register of Dams (WRD), the International Commission on Large Dams (ICOLD; https://www.icold-cigb.org; last access on March 13th, 2019).
- **GeoDAR_v11_dams** (in both shapefile and csv): GeoDAR version 1.1 dam points, including 24,783 dams which harmonized GeoDAR_v10_dams and the Global Reservoir and Dam Database (GRanD) v1.3 (Lehner et al., 2011).
- **GeoDAR_v11_reservoirs** (in shapefile): GeoDAR version 1.1 reservoirs, including 21,515 reservoir polygons retrieved by associating GeoDAR_v11_dams with GRanD v1.3 reservoirs, HydroLAKES v1.0 (Messager et al., 2016), and the UCLA Circa 2015 Lake Inventory (Sheng et al., 2016). The reservoir retrieval follows a one-to-one relationship between dams and reservoirs.

As by-products of GeoDAR harmonization, we also provide:

- **GRanD_v13_issues.csv**: This file contains the original records of all 7,320 dam points in GRanD v1.3, with 94 of them marked by our identified issues and suggested corrections. These 94 records are placed at the beginning of this table. They include 89 records showing possible georeferencing and/or attribute errors, and another 5 records documented as subsumed or replaced. Our added fields start from column BG and include:
    - "Issue": main issue(s) of this record

    - "Description": more detailed explanation of the issue
    - "Lat_corrected": suggested correction for latitude (if any) in decimal degree
    - "Lon_corrected": suggested correction for longitude (if any) in decimal degree
    - "Correction_source": correction source(s)
    - "Harmonized": whether this GRanD dam was harmonized in GeoDAR v1.1 and the reason.
- **Wada_et_al_2017_harmonized.csv**: This csv file contains the original records of all 139 georeferenced large dams/reservoirs in Wada et al. (2017), with our revised storage capacities and spatial coordinates for data harmonization. Our added fields start from column E and include:
    - Revised_capacity_km3: Our revised reservoir storage capacity in cubic kilometers used for harmonization
    - Revised_lat: Revised latitude in decimal degree
    - Revised_lon: Revised longitude in decimal degree
    - Verification_notes: Description of the issues, verification sources, and other information used for harmonization.

Attribute description

Attribute	Description and values
**v1.0 dams (file name: GeoDAR_v10_dams; format: comma-separated values (csv) and point shapefile)**	
id_v10	Dam ID for GeoDAR version 1.0 (type: integer). Note this is not the same as the International Code in ICOLD WRD but is linked to the International Code via encryption.
lat	Latitude of the dam point in decimal degree (type: float) based on datum World Geodetic System (WGS) 1984.
lon	Longitude of the dam point in decimal degree (type: float) on WGS 1984.
geo_mtd	Georeferencing method (type: text). Unique values include "geo-matching CanVec", "geo-matching LRD", "geo-matching MARS", "geo-matching NID", "geo-matching ODC", "geo-matching ODM", "geo-matching RSB", "geocoding (Google Maps)", and "Wada et al. (2017)". Refer to Table 2 in Wang et al. (2022) for abbreviations.
qa_rank	Quality assurance (QA) ranking (type: text). Unique values include "M1", "M2", "M3", "C1", "C2", "C3", "C4", and "C5". The QA ranking provides a general measure for our georeferencing quality. Refer to Supplementary Tables S1 and S3 in Wang et al. (2022) for more explanation.
rv_mcm	Reservoir storage capacity in million cubic meters (type: float). Values are only available for large dams in Wada et al. (2017). Capacity values of other WRD records are not released due to ICOLD's proprietary restriction. Also see Table S4 in Wang et al. (2022).
val_scn	Validation result (type: text). Unique values include "correct", "register", "mismatch", "misplacement", and "Google Maps". Refer to Table 4 in Wang et al. (2022) for explanation.
val_src	Main validation source(s) (type: text). Values include "CanVec", "Google Maps", "JDF", "LRD", "MARS", "NID", "NPCGIS", "NRLD", "ODC", "ODM", "RSB", "Wada et al. (2017)", and other miscellaneous references. Refer to Table 2 in Wang et al. (2022) for abbreviations.
qc	Roles and name initials of co-authors/participants during data quality control (QC) and validation. Name initials are given to each assigned dam or region and are listed generally in chronological order for each role. Collation and harmonization of large dams in Wada et al. (2017) (see Table S4 in Wang et al. (2022)) were performed by JW, and this information is not repeated in the *qc* attribute for a reduced file size. Although we tried to track the name initials thoroughly, the lists may not be always exhaustive, and other undocumented adjustments and corrections were most likely performed by JW.

**v1.1 dams (file name: GeoDAR_v11_dams; format: comma-separated values (csv) and point shapefile)**	
id_v11	Dam ID for GeoDAR version 1.1 (type: integer). Note this is not the same as the International Code in ICOLD WRD but is linked to the International Code via encryption.
id_v10	v1.0 ID of this dam/reservoir (as in *id_v10*) if it is also included in v1.0 (type: integer).
id_grd_v13	GRanD ID of this dam if also included in GRanD v1.3 (type: integer).
lat	Latitude of the dam point in decimal degree (type: float) on WGS 1984. Value may be different from that in v1.0.
lon	Longitude of the dam point in decimal degree (type: float) on WGS 1984. Value may be different from that in v1.0.
geo_mtd	Same as the value of *geo_mtd* in v1.0 if this dam is included in v1.0.
qa_rank	Same as *qa_rank* in v1.0 if this dam is included in v1.0.
val_scn	Same as *val_scn* in v1.0 if this dam is included in v1.0.
val_src	Same as *val_src* in v1.0 if this dam is included in v1.0.
rv_mcm_v10	Same as *rv_mcm* in v1.0 if this dam is included in v1.0.
rv_mcm_v11	Reservoir storage capacity in million cubic meters (type: float). Due to ICOLD's proprietary restriction, provided values are limited to dams in Wada et al. (2017) and GRanD v1.3. If a dam is in both Wada et al. (2017) and GRanD v1.3, the value from the latter (if valid) takes precedence.
har_src	Source(s) to harmonize the dam points. Unique values include "GeoDAR v1.0 alone", "GRanD v1.3 and GeoDAR 1.0", "GRanD v1.3 and other ICOLD", and "GRanD v1.3 alone". Refer to Table 1 in Wang et al. (2022) for more details.
pnt_src	Source(s) of the dam point spatial coordinates. Unique values include "GeoDAR v1.0", "original GRanD", "adjusted GRanD" (meaning the original dam point location in GRanD has been adjusted to improve the accuracy), and "corrected GRanD" (meaning the original point in GRanD was misplaced and has been corrected). Also see Table S5 in Wang et al. (2022).
qc	Roles and name initials of co-authors/participants during data QC, validation, and other manual operations. Name initials are given to each assigned dam or region and are listed generally in chronological order for each role. Correction of GRanD (see Table S5 in Wang et al. (2022)) and reservoir polygon QC were performed by JW, and this information is not repeated in the *qc* attribute to reduce the file size. Although we tried to track the name initials thoroughly, the lists may not be always exhaustive, and other undocumented adjustments and corrections were most likely performed by JW.
**v1.1 reservoirs (file name: GeoDAR_v11_reservoirs; format: polygon shapefile)**	
plg_src	Source of the retrieved reservoir polygon (type: text). Unique values include "GRanD v1.3", "HydroLAKES v1.0", and "UCLA Circa 2015". Refer to Table 1 in Wang et al. (2022) for more details.
plg_a_km2	Area of the retrieved reservoir polygon in square kilometres (calculated based on the cylindrical equal area projection on datum WGS 1984).
All other attributes in v1.1 dams.	

Note: Missing or inapplicable values are flagged by "-999" for numeric-type attributes.

Data and code availability

GeoDAR v1.0 (dam points) and v1.1 (both dam points and reservoir polygons) are available under the Creative Commons Attribution 4.0 International (CC-BY 4.0) license (https://creativecommons.org/licenses/by/4.0).

Any user who would like to link GeoDAR features to the proprietary WRD attributes the user has purchased in advance from ICOLD should contact the corresponding author JW.

Python scripts for geo-matching, geocoding, and reservoir assignment are available at https://github.com/surf-hydro/georeferencing-ICOLD-dams-and-reservoirs. We request users who adapt or use the scripts to cite Wang et al. (2022).

We also request users to cite Wang et al. (2022) if they use our identified issues or suggested corrections for GRanD v1.3 (as provided in “GRanD_v13_issues.csv”).

Disclaimer

GeoDAR v1.0 and v1.1 contain knowledge derived from ICOLD WRD (https://www.icold-cigb.org/GB/world_register/acknowledgements_wrd.asp) but release no original values of the proprietary WRD attributes (except the storage capacities of a few large dams used to verify/correct Wada et al. (2017); see Table S4 in Wang et al. (2022)). The production and dissemination of GeoDAR abide by ICOLD’s legal policies (https://www.icold-cigb.org/GB/legal.asp) and were approved by the central office of ICOLD.

GeoDAR v1.0 represents an initial effort of georeferencing WRD at the global scale. The resultant dam distribution may be skewed towards regions where georeferencing sources are more abundant, and therefore, may not accurately reflect the distribution of all WRD records. In addition, since we leveraged multiple data sources and methods to georeference the WRD records, the produced "dam" points in both versions do not always fall exactly on the dam bodies and instead, are often located on the surface of the associated reservoirs. In our QC, we manually adjusted some of the points to be closer to the dams, but more improvement can still be made. The authors are not responsible for any consequence arising from these limitations.

GeoDAR v1.1 absorbed most of the spatial features (i.e., dam point coordinates and reservoir polygons) in GRanD v1.3. To acknowledge the originality of GRanD, we request users to cite Lehner et al. (2011) if they only use the subset of GeoDAR v1.1 from GRanD alone. If the user adopts the spatial coordinates we corrected for GRanD (see “GRanD_v13_issues.csv”), we recommend users citing Wang et al. (2022) as well.

The source of each spatial feature in GeoDAR v1.1 is specified in the attributes “har_src” and “pnt_src” for dam points and the attribute “plg_src” for reservoir polygons. For any questions about data citation, please contact the corresponding author JW.

Authors of this paper claim no responsibility or liability for any consequences related to the use, citation, or dissemination of GeoDAR.

Citation

Wang, J., Walter, B. A., Yao, F., Song, C., Ding, M., Maroof, A. S., Zhu, J., Fan, C., McAlister, J. M., Sikder, M. S., Sheng, Y., Allen, G. H., Crétaux, J.-F., and Wada, Y.: GeoDAR: georeferenced global dams and reservoirs database for bridging attributes and geolocations. Earth System Science Data, 14, 1-31, 2022, doi: 10.5194/essd-14-1-2022.

References

Lehner, B., Liermann, C. R., Revenga, C., Vörösmarty, C., Fekete, B., Crouzet, P., Döll, P., Endejan, M., Frenken, K., Magome, J., Nilsson, C., Robertson, J. C., Rödel, R., Sindorf, N., and Wisser, D.: High-resolution mapping of the world’s reservoirs and dams for sustainable river-

flow management, Frontiers in Ecology and the Environment, 9, 494-502, 2011, doi: 10.1890/100125.

Messager, M. L., Lehner, B., Grill, G., Nedeva, I., and Schmitt, O.: Estimating the volume and age of water stored in global lakes using a geo-statistical approach, Nature Communications, 7, 13603, 2016, doi: 10.1038/ncomms13603.

Sheng, Y., Song, C., Wang, J., Lyons, E. A., Knox, B. R., Cox, J. S., and Gao, F.: Representative lake water extent mapping at continental scales using multi-temporal Landsat-8 imagery, Remote Sensing of Environment, 185, 129-141, 2016, doi: 10.1016/j.rse.2015.12.041.

Wada, Y., Reager, J. T., Chao, B. F., Wang, J., Lo, M.-H., Song, C., Li, Y., and Gardner, A. S.: Recent changes in land water storage and its contribution to sea level variations, Surveys in Geophysics, 38, 131-152, 2017, doi: 10.1007/s10712-016-9399-6.

Wang, J., Walter, B. A., Yao, F., Song, C., Ding, M., Maroof, A. S., Zhu, J., Fan, C., McAlister, J. M., Sikder, M. S., Sheng, Y., Allen, G. H., Crétaux, J.-F., and Wada, Y.: GeoDAR: georeferenced global dams and reservoirs database for bridging attributes and geolocations. Earth System Science Data, 14, 1-31, 2022, doi: 10.5194/essd-14-1-2022.
