# Supplementary material for: How Marine Megabenthos Fauna Responds to River Discharge and Artificial Flood in Large River Estuary
Source: Ecol Evol. 2025 Jan 8;15(1):e70755. doi: 10.1002/ece3.70755 (PMC11707399; doi:10.1002/ece3.70755)
Supplement: Supplementary file 4 — Figure S1. The data statistics of C‐diversity the ACA for three cruises (IA, IB, and IC). (A) Variation of the C‐diversity index at a distance from the estuary. B: Comparison of C‐diversity index in different IA, B, C periods. (C) Comparison of C‐diversity in the estuary region (E), the transition region (M), and the region staying away from the estuary (F). (D) C‐diversity of estuary region in different periods. * denotes a significant difference between the two regions (p < 0.05). Figure S2. The leading phylogenetic diversity indices of the megabenthos community. PD, PSR, PSE, PSV, MPD, and MNTD denote the phylogenetic diversity, phylogenetic species richness, phylogenetic species evenness, phylogenetic species variability, mean pairwise distance, and mean nearest taxon distance, respectively. *denotes a significant difference between the two regions (p < 0.05). Figure S3. The stability indices of megabenthic community. AVD, ICV, C_pos, Robustness_R, Robustness_Y, and Vulnerability denote the average variation degree, community stability index, community positive cohesion, network robustness (the proportion of species remaining after 50% of the species are randomly removed from each community), network robustness (the proportion of species remaining after the dominant species are removed from each community), and community vulnerability. *denotes a significant difference between the two regions (p < 0.05). Figure S4. Pearson’s analysis of environmental parameters (left) and Mantel’s test among dominant taxa of megabenthos with environmental parameters (right) based on pooled abundance data. Note: Major taxa of megabenthos denote major family groups with more than 10% of the species occurrence frequency in pooled abundance data. Figure S5. Network analysis for the co‐occurrence of megabenthos in the estuarine community in each period (IA, IB, and IC) based on the relative abundance. The (d, l, c, m) denote the average degree, average path length, clustering coeffic [file ECE3-15-e70755-s004.docx]

# How marine megabenthos fauna responds to river discharge and artificial flood in large river estuary

**Supplementary Information**

**Contents**

**1. Supplementary Figures 1-5**

**2. Supplementary Tables 1-6**

**3. Supplementary Texts A-C**

**Supplementary Figures 1-5**


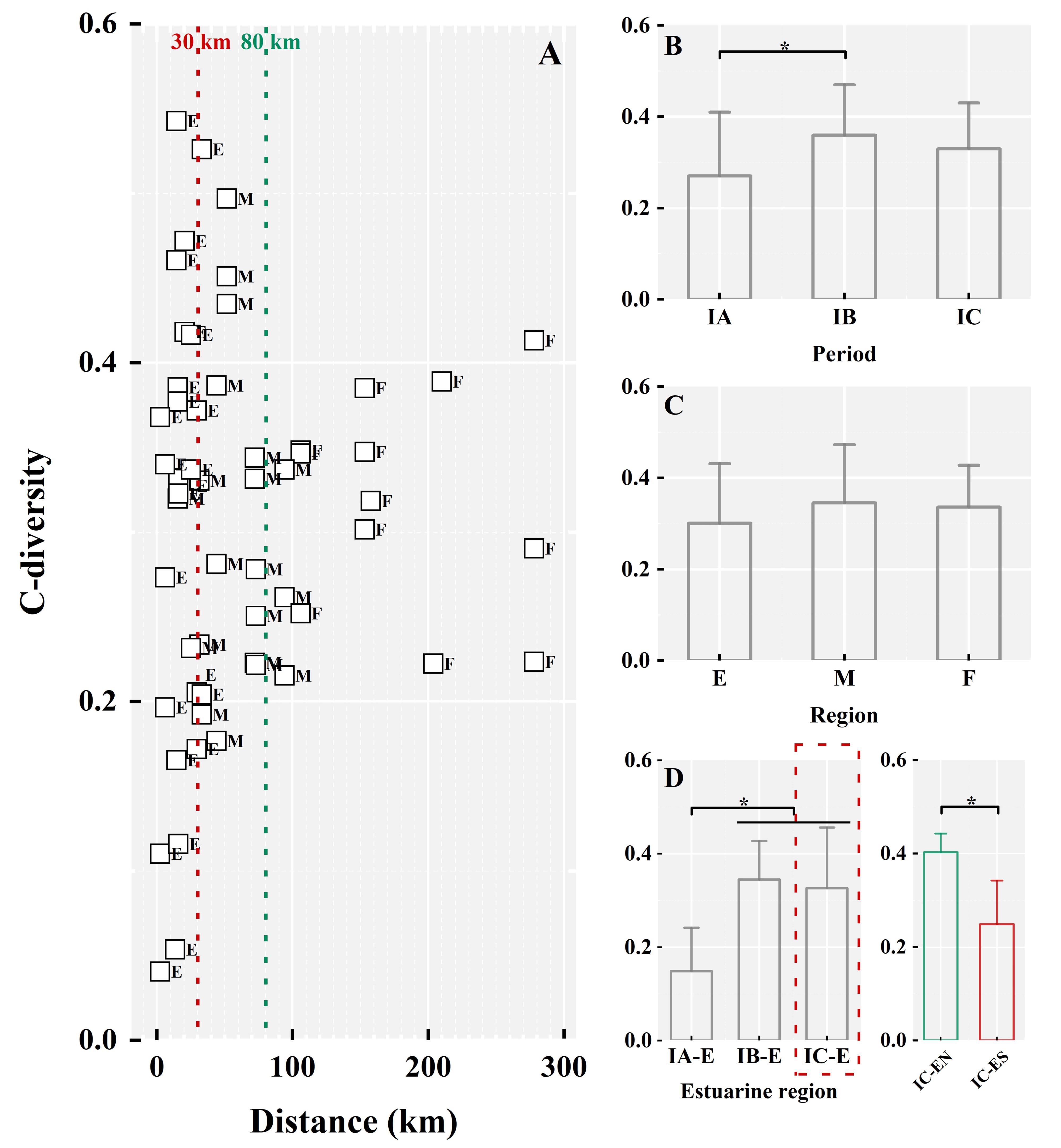


**Sup Fig. 1** The data statistics of *C-diversity* the ACA for three cruises (IA, IB, and IC). A: Variation of the *C-diversity* index at a distance from the estuary. B: Comparison of *C-diversity* index in different IA, IB, and IC periods. C: Comparison of *C-diversity* in the estuary region (E), the transition region (M), and the region staying away from the estuary (F). D: *C-diversity* of estuary region in different periods. The * denotes a significant difference between the two regions (*p* < 0.05).


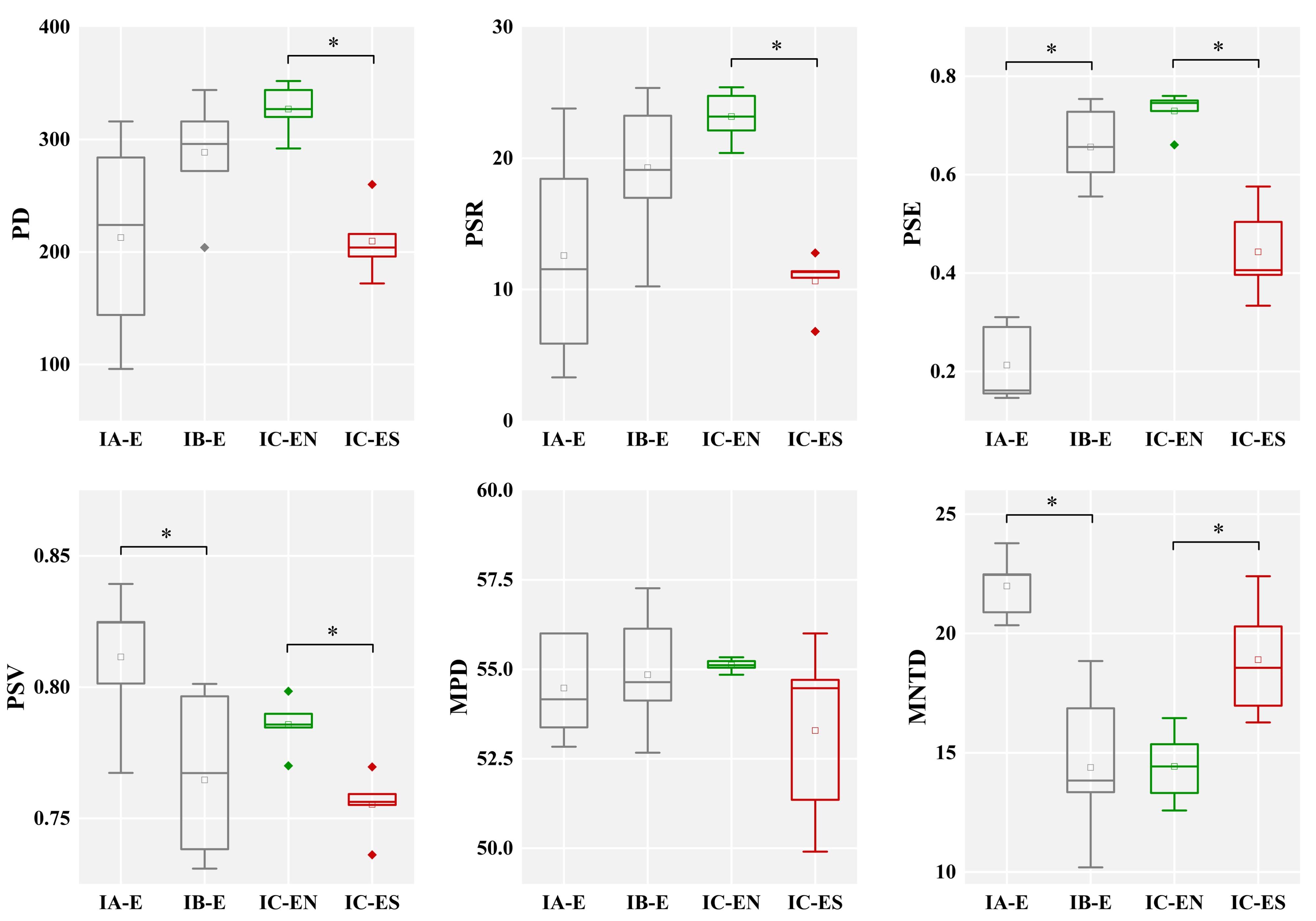


**Sup Fig. 2** The leading phylogenetic diversity indices of the megabenthos community. *PD*, *PSR*, *PSE*, *PSV*, *MPD*, and *MNTD* denote the phylogenetic diversity, phylogenetic species richness, phylogenetic species evenness, phylogenetic species variability, mean pairwise distance, and mean nearest taxon distance, respectively. The * denotes a significant difference between the two regions (*p* < 0.05).


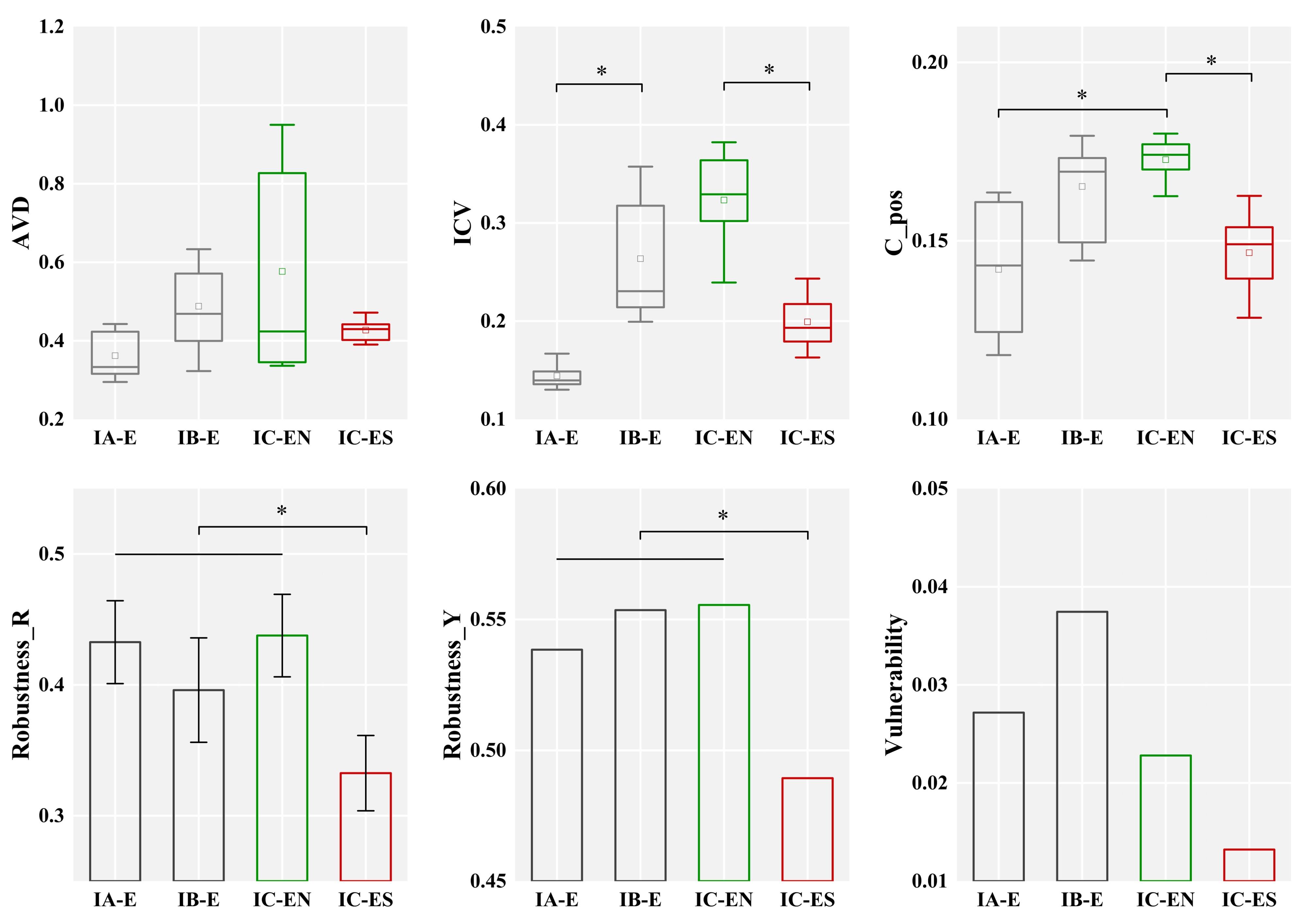


**Sup Fig. 3** The stability indices of megabenthic community. *AVD*, *ICV*, *C_pos*, *Robustness_R*, *Robustness_Y*, and *Vulnerability* denote the average variation degree, community stability index, community positive cohesion, network robustness (the proportion of species remaining after 50% of the species are randomly removed from each community), network robustness (the proportion of species remaining after the dominant species are removed from each community), and community vulnerability. The * denotes a significant difference between the two regions (*p* < 0.05).


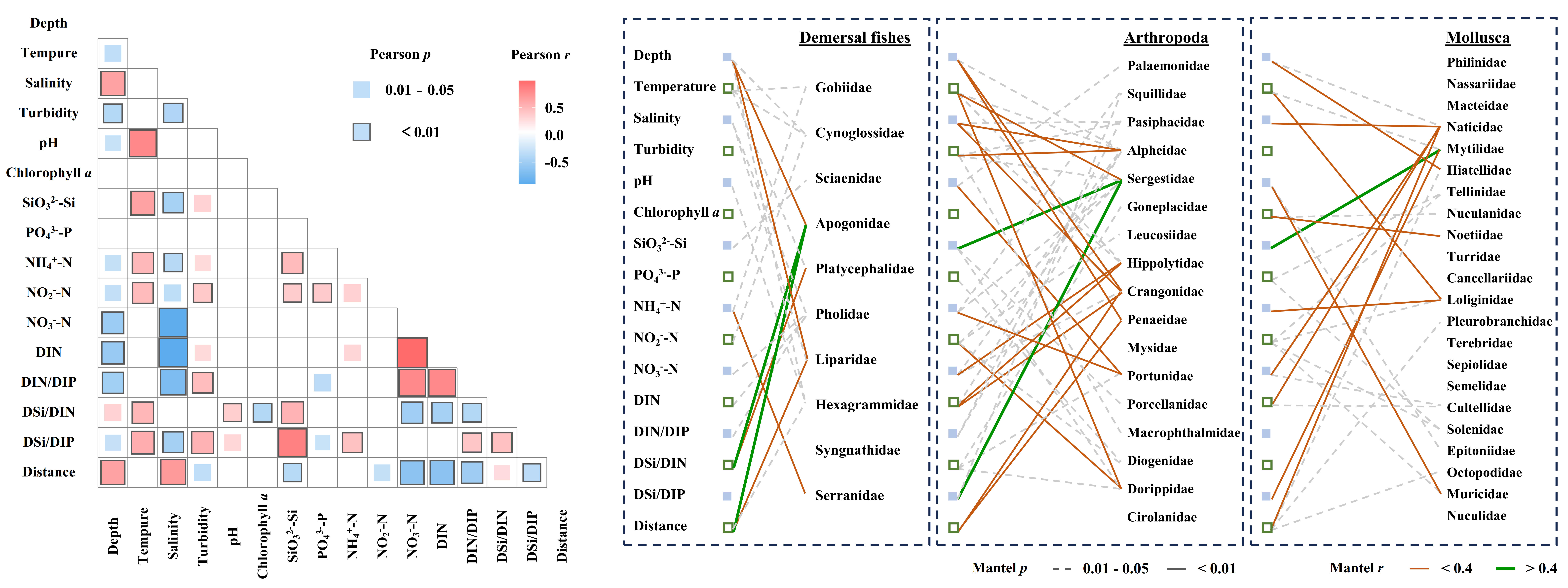


**Sup Fig. 4** Pearson’s analysis of environmental parameters (left) and Mantel’s test among dominant taxa of megabenthos with environmental parameters (right) based on pooled abundance data. Notes: Major taxa of megabenthos denote major family groups with more than 10% of the species occurrence frequency in pooled abundance data.


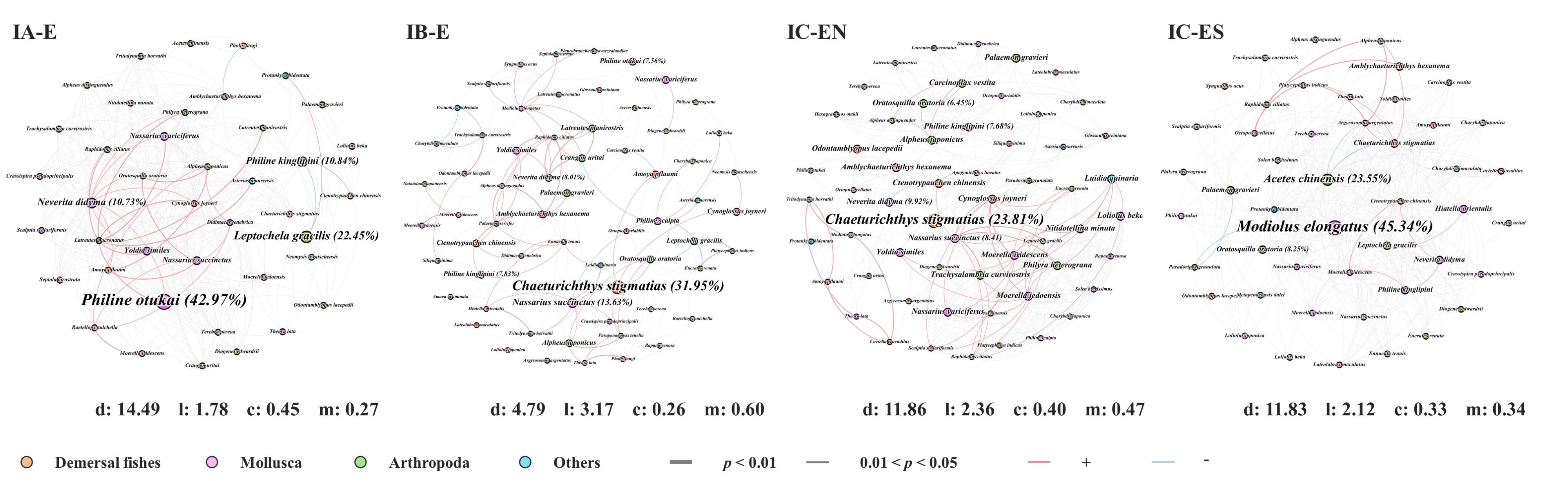


**Sup Fig. 5** Network analysis for the co-occurrence of megabenthos in the estuarine community in each period (IA, IB, and IC) based on the relative abundance. The d, l, c, and m denote the average degree, average path length, clustering coefficient, and modularity index of the network, respectively.

**Supplementary Tables 1-6**

**Sup Table 1:** Overview of different indices of megabenthos community employed in this study.

|  | Index | Formula | | Citation |
| --- | --- | --- | --- | --- |
| Community biodiversity | Species richness | *S* | (1) | \ |
|  | Species abundance | *S_a_ = log(x_i_+1)* | (2) | \ |
|  | Species biomass | *S_b_ = log(x_i_+1)* | (3) | \ |
|  | Shannon-Wiener diversity index  (*H´*) | $\text{H´ =}\text{ }\sum_{\text{i=1}}^{\text{ s}} \text{P}_{\text{i}}\text{lnP}_{\text{i}}$ | (4) | Shannon, C E, et al., 1949 |
|  | Margalef species richness index  (*d*) | $\text{d}\text{ =}\text{ }\frac{\text{S-1}}{\text{lnN}}$ | (5) | Margalef, R A, 1951 |
|  | Pielou species evenness index  (*J*´) | $\text{J´}\text{ }\text{=}\text{ }\text{ }\frac{\text{ }\text{H´}}{\text{lnS}}$ | (6) | Pielou, E C, 1966 |
|  | Simpson diversity index  (*D*) | $\text{D = 1 - }\sum_{\text{i=1}}^{\text{ S}} {\text{(}\text{P}_{\text{i}}\text{)}}^{\text{2}}$ | (7) | Simpson, E H, 1949 |
|  | Inverse of Simpson index  (*D*´) | $\text{D´ = 1/ }\sum_{\text{i=1}}^{\text{ }\text{S}} {\text{(}\text{P}_{\text{i}}\text{)}}^{\text{2}}$ | (8) | Simpson, E H, 1949 |
|  |  |  |  |  |
| Community phylogenetic diversity | Phylogenetic diversity  (*PD*) | $\text{PD = }\sum_{\text{i=1}}^{\text{ N}} \text{L}_{\text{i}}$ | (9) | Faith, D P, 1992 |
|  | Mean pairwise distance  (*MPD*) | $\text{MPD}\text{ = }\frac{\sum\sum_{\text{i≠j}} \text{P}\text{D}_{\text{i,j}}}{\text{n(n-1)/2}}$ | (10) | O, W C, 2000 |
|  | Mean nearest taxon distance  (*MNTD*) | $\text{MNTD = }\frac{\sum_{\text{i=1}}^{\text{ n}} \text{minP}\text{D}_{\text{i}}}{\text{n}}$ | (11) | O, W C, 2000 |
|  | Nearest relative index  (*NRI*) | $\text{NRI = }\frac{\overline{\text{d}} \text{-}\text{ }\overline{\text{r}}}{\text{SD(r)}}$ | (12) | O, W C, 2000 |
|  | Phylogenetic species variability  (*PSV*) | $\text{PSV =}\frac{\text{ntr}\text{C}\text{ -}\sum\text{C}}{\text{n(n-1)}}$ | (13) | R, H M, et al., 2007 |
|  | Phylogenetic species evenness  (*PSE*) | $\text{PSE = }\frac{\text{mdiag(}\text{C}\text{)}\text{´}\text{M}\text{ - }\text{M}\text{´}\text{CM}}{\text{m}^{\text{2}} \text{-}\text{ }\overline{\text{m}_{\text{i}}}\text{m}}$ | (14) | R, H M, et al., 2007 |
|  | Phylogenetic species richness  (*PSR*) | $\text{PSR =}\text{ }\text{nPSV}$ | (15) | R, H M, et al., 2007 |
|  | Phylogenetic species clustering  (*PSC*) | $\text{PSC = 1 - }\frac{\sum_{\text{i=1}}^{\text{ n}} \text{max}\text{(}\text{c}_{\text{i-}}\text{)}}{\text{n}}$ | (16) | R, H M, et al., 2007 |
|  |  |  |  |  |
| Community stability | Community stability index  (*ICV*) | $\text{ICV = μ/σ}$ | (18) | Tilman, D, 1999 |
|  | Positive cohesion  (*C_pos*) | $\text{C}_{\text{\_pos}}\text{ = }\sum_{\text{i=1}}^{\text{n}} \text{a}_{\text{i}}{\overline{\text{r}}}_{\text{i,r>0}}$ | (19) | Herren, C M, et al., 2017 |
|  | Negative cohesion  (*C_neg*) | $\text{C}_{\text{\_}\text{neg}}\text{ = }\sum_{\text{i=1}}^{\text{n}} \text{a}_{\text{i}}{\overline{\text{r}}}_{\text{i,r}\text{<}\text{0}}$ | (20) | Herren, C M, et al., 2017 |
|  | Total cohesion  (*C_total*) | $\text{C}_{\text{\_total}}\text{ =}\text{ C}_{\text{\_pos}}\text{ +}\text{ }\left\vert\text{C}_{\text{\_neg}} \right\vert$ | (21) | Hernandez, D J, et al., 2021 |
|  | Average variation degree  (*AVD*) | $\text{AVD = }\frac{\sum_{\text{i=1}}^{\text{ n}} \frac{\left\vert\text{x}_{\text{i}}\text{-}{\overline{\text{x}}}_{\text{i}} \right\vert}{\text{δ}_{\text{i}}}}{\text{k × n}}$ | (17) | Weibing, X, et al., 2021 |
|  | Robustness for random species removal (*Robustness_R*) | $\text{wMIS}_{\text{i}}\text{ = }\frac{\sum_{\text{j≠i}} \text{b}_{\text{j}}\text{s}_{\text{ij}}}{\sum_{\text{j≠i}} \text{bj}}$  $\text{Robustness\_R}\text{ = 1 - }\frac{\text{N}_{\text{ex}}}{\text{N}}$ | (22) | Yuan, M M, et al., 2021 |
|  | Robustness for dominant species removal  (*Robustness_Y*) | $\text{wMIS}_{\text{i}}\text{ = }\frac{\sum_{\text{j≠i}} \text{b}_{\text{j}}\text{s}_{\text{ij}}}{\sum_{\text{j≠i}} \text{bj}}$  $\text{Robustness\_}\text{Y}\text{ = 1 - }\frac{\text{N}_{\text{ex}}}{\text{N}}$ | (23) | Yuan, M M, et al., 2021 |
|  | Vulnerability metrics  (*Vulnerability*) | $\text{E = }\frac{\text{1}}{\text{n(n-1)}}\text{ }\sum_{\text{i≠j}} \frac{\text{1}}{\text{d}_{\text{ij}}}$  $\text{Vulnerability = max(}\frac{\text{E-}\text{E}_{\text{i}}}{\text{E}}\text{)}$ | (24) | Yuan, M M, et al., 2021 |
|  |  |  |  |  |
| Comprehensive index | Comprehensive biodiversity  (*C-diversity*) | $\text{C-diversity = }\sum_{\text{j=1}}^{\text{ m}} \text{W}_{\text{j}}\text{y}_{\text{ij}}$  $\text{(j: indices of 1-8 in t}\text{h}\text{is paper)}$ | (25) | This paper |
|  | Comprehensive phylogenetic diversity  (*C-phyl*) | $\text{C-}\text{phyl}\text{ = }\sum_{\text{j=1}}^{\text{ m}} \text{W}_{\text{j}}\text{y}_{\text{ij}}$  $\text{(j: indices of 9-16 in t}\text{h}\text{is paper)}$ | (26) | This paper |
|  | Comprehensive stability  (C-*stability*) | $\text{C-stability = }\sum_{\text{j=1}}^{\text{ m}} \text{W}_{\text{j}}\text{y}_{\text{ij}}$  $\text{(j: indices of }\text{17}\text{-}\text{24}\text{ in t}\text{h}\text{is paper)}$ | (27) | This paper |

**Sup Table 2** The weight and mean values (mean ± sd) of the comprehensive biodiversity index (*C-diversity*) for three cruises (IA, IB, and IC).

| **Community Indices** |  | **IA** | **IB** | **IC** |  | **Entropy Weight** |
| --- | --- | --- | --- | --- | --- | --- |
| ***S_b_*** |  | **1.91±2.08** | **2.25±2.16** | **2.12±2.13** |  | **0.29** |
| ***S_a_*** |  | **4.72±4.81** | **5.05±5.01** | **4.88±4.85** |  | **0.27** |
| ***D´*** |  | **5.10±3.28** | **4.40±2.47** | **4.93±2.38** |  | **0.16** |
| ***S*** |  | **22.37±10.30** | **28.79±7.76** | **24.78±10.38** |  | **0.07** |
| ***H´*** |  | **1.83±0.73** | **1.75±0.71** | **1.94±0.45** |  | **0.06** |
| ***D*** |  | **0.69±0.24** | **0.66±0.25** | **0.74±0.13** |  | **0.05** |
| ***J´*** |  | **0.61±0.22** | **0.52±0.21** | **0.63±0.15** |  | **0.05** |
| ***d*** |  | **2.06±0.89** | **2.45±0.6** | **2.17±0.80** |  | **0.05** |
| ***C-diversity*** |  | **0.27±0.14** | **0.36±0.11** | **0.33±0.10** |  |  |
|  |  |  |  |  |  |  |

**Sup Table 3** The weight and mean values (mean ± sd) of the comprehensive index of phylogenetic diversity (*C-phyl*) for three cruises (IA, IB, and IC).

| **Community Indices** |  | **IA** | **IB** | **IC** |  | **Entropy Weight** |
| --- | --- | --- | --- | --- | --- | --- |
| ***PSR*** |  | **13.65±5.84** | **18.63±4.92** | **16.21±5.84** |  | **0.20** |
| ***PSE*** |  | **0.53±0.21** | **0.54±0.22** | **0.59±0.17** |  | **0.19** |
| ***NRI*** |  | **-0.16±0.67** | **-0.62±0.74** | **-0.44±0.65** |  | **0.14** |
| ***PD*** |  | **231.2±60.38** | **283.2±46.89** | **260.58±56.54** |  | **0.13** |
| ***MNTD*** |  | **17.33±5.21** | **15.17±4.5** | **15.73±2.84** |  | **0.09** |
| ***PSC*** |  | **0.29±0.06** | **0.24±0.04** | **0.27±0.05** |  | **0.09** |
| ***MPD*** |  | **54.07±2.52** | **54.88±1.64** | **54.35±1.89** |  | **0.08** |
| ***PSV*** |  | **0.79±0.05** | **0.77±0.02** | **0.77±0.03** |  | **0.07** |
| ***C-phyl*** |  | **0.56±0.13** | **0.68±0.12** | **0.64±0.16** |  |  |
|  |  |  |  |  |  |  |

**Sup Table 4** The weight and mean values (mean ± sd) of the comprehensive index of stability (*C-stability*) for three cruises (IA, IB, and IC).

| **Community Indices** |  | **IA** | **IB** | **IC** |  | **Entropy Weight** |
| --- | --- | --- | --- | --- | --- | --- |
| ***C_neg*** |  | **-0.14±0.01** | **-0.16±0.01** | **-0.13±0.01** |  | **0.28** |
| ***Robustness_R*** |  | **0.32** | **0.38** | **0.38** |  | **0.13** |
| ***Robustness_Y*** |  | **0.54** | **0.51** | **0.50** |  | **0.13** |
| ***Vulnerability*** |  | **0.01** | **0.04** | **0.03** |  | **0.11** |
| ***ICV*** |  | **0.25±0.08** | **0.23±0.08** | **0.25±0.07** |  | **0.10** |
| ***C_total*** |  | **0.29±0.02** | **0.29±0.02** | **0.29±0.02** |  | **0.09** |
| ***AVD*** |  | **0.43±0.14** | **0.52±0.19** | **0.54±0.22** |  | **0.09** |
| ***C_pos*** |  | **0.15±0.02** | **0.16±0.02** | **0.16±0.02** |  | **0.08** |
| ***C-stability*** |  | **0.41** | **0.56** | **0.46** |  |  |
|  |  |  |  |  |  |  |

**Sup Table 5** Ranges, mean values (± sd), and variation coefficient values (CV) of environmental parameters in the bottom seawater layers during IA, IB, and IC period.

| **Parameters** | **IA** | |  | **IB** | |  | **IC** | |
| --- | --- | --- | --- | --- | --- | --- | --- | --- |
|  | **Range** | **Mean±sd** |  | **Range** | **Mean±sd** |  | **Range** | **Mean±sd** |
| **Sa (‰)** | **24.25-31.57** | **28.68±2.02** |  | **24.06-31.38** | **29.07±1.75** |  | **22.15-30.71** | **28.79±1.77** |
| **Tu (NTU)** | **0.90-35.96** | **7.62±8.44** |  | **0.40-21.16** | **4.49±3.90** |  | **1.52-45.82** | **12.15±11.40** |
| **Tem (℃)** | **9.02-16.04** | **12.52±1.52** |  | **11.53-23.45** | **17.57±3.11** |  | **18.36-28.16** | **23.26±2.93** |
| **pH** | **8.19-8.44** | **8.35±0.04** |  | **8.38-8.65** | **8.49±0.06** |  | **8.31-8.65** | **8.51±0.08** |
| **Chl (μg·L^-1^)** | **1.06-4.20** | **2.14±0.72** |  | **1.23-6.82** | **3.11±1.38** |  | **0.37-5.93** | **1.75±1.01** |
| **NH_4_^+^-N (mg·L^-1^)** | **0.01-0.11** | **0.03±0.02** |  | **0.01-0.09** | **0.03±0.02** |  | **0.02-0.20** | **0.07±0.04** |
| **NO_3_^-^-N (mg·L^-1^)** | **0.01-0.87** | **0.34±0.25** |  | **0.01-0.58** | **0.22±0.16** |  | **0.01-0.68** | **0.15±0.13** |
| **NO_2_^-^-N (ug·L^-1^)** | **1.20-12.10** | **4.56±2.38** |  | **0.50-13.30** | **4.18±2.29** |  | **1.70-64.50** | **9.71±11.14** |
| **DIN (mg·L^-1^)** | **0.02-0.93** | **0.37±0.26** |  | **0.01-0.65** | **0.26±0.17** |  | **0.03-0.84** | **0.23±0.15** |
| **PO_4_^3-^-P (ug·L^-1^)** | **1.90-12.20** | **5.98±1.59** |  | **1.70-11.30** | **4.61±1.59** |  | **2.10-10.90** | **5.53±1.95** |
| **SiO_3_^2-^-Si (mg·L^-1^)** | **0.01-0.30** | **0.10±0.07** |  | **0.02-0.20** | **0.09±0.05** |  | **0.09-0.95** | **0.31±0.18** |
| **DIN/DIP** | **6.99-819.42** | **152.64±138.15** |  | **11.88-599.60** | **131.60±101.12** |  | **9.05-318.62** | **96.58±59.71** |
| **DSi/DIN** | **0.04-0.96** | **0.16±0.13** |  | **0.06-0.78** | **0.24±0.15** |  | **0.24-1.73** | **0.80±0.37** |
| **DSi/DIP** | **1.22-119.65** | **20.87±19.64** |  | **2.40-65.01** | **23.33±12.58** |  | **15.65-179.75** | **64.55±33.33** |

**Sup Table 6:** The dominant species and dominance degree (*Y*) of the estuarine community in each period (IA, IB, and IC).

| **IA-E** | |  | **IB-E** | |  | **IC-ES** | |  | **IC-ES** | |
| --- | --- | --- | --- | --- | --- | --- | --- | --- | --- | --- |
| **Species** | ***Y*** |  | **Species** | ***Y*** |  | **Species** | ***Y*** |  | **Species** | ***Y*** |
| ***L. gracilis*** | **0.18** |  | ***C. stigmatias*** | **0.28** |  | ***C. stigmatias*** | **0.17** |  | ***M. elongatus*** | **0.36** |
| ***P. otukai*** | **0.17** |  | ***N. succinctus*** | **0.19** |  | ***N. succinctus*** | **0.08** |  | ***A. chinensis*** | **0.19** |
| ***N. didyma*** | **0.09** |  | ***N. didyma*** | **0.11** |  | ***O. oratoria*** | **0.07** |  | ***O. oratoria*** | **0.07** |
| ***P. kinglipini*** | **0.04** |  | ***L. gracilis*** | **0.06** |  | ***N. didyma*** | **0.05** |  | ***P. gravieri*** | **0.04** |
| ***N. succinctus*** | **0.02** |  | ***P. otukai*** | **0.05** |  | ***C. joyneri*** | **0.04** |  |  |  |
|  |  |  | ***A. japonicus*** | **0.04** |  | ***P. gravieri*** | **0.04** |  |  |  |
|  |  |  | ***P. kinglipini*** | **0.04** |  | ***A. argentatus*** | **0.03** |  |  |  |
|  |  |  | ***C. chinensis*** | **0.03** |  | ***M. jedoensis*** | **0.02** |  |  |  |
|  |  |  | ***A. hexanema*** | **0.03** |  | ***M. iridescens*** | **0.02** |  |  |  |
|  |  |  | ***N. variciferus*** | **0.02** |  |  |  |  |  |  |

**Supplementary Texts A-C**

**A. The calculation statements of each index in Sup Table 1.**

The species richness (*S*), species abundance (*Sa*), species biomass (*Sb*), and other sample characteristics were recorded and transformed by the *R* language. Shannon-Wiener diversity index (*H´*), Margalef species richness index (*d*), Pielou species evenness index (*J´*), Simpson diversity index (*D*), and inverse of Simpson index (*D´*) were calculated in the package {picante}. The phylogenetic information was obtained at NCBI (https://www. ncbi.nlm.nih.gov/). Phylogenetic diversity (*PD*), mean pairwise distance (*MPD*), mean nearest taxon distance (*MNTD*), nearest relative index, phylogenetic species variability (*NRI*), phylogenetic species evenness (*PSV*), phylogenetic species richness (*PSR*), and phylogenetic species clustering (*PSC*) were calculated in package {picante} and package {ape} (TEICHERT, N, et al., 2018; JIAO, W, et al., 2023). The community stability index (*ICV*) and average variation degree (*AVD*) were calculated by the formula in **Sup Table 1**. The positive cohesion (*C_pos*), negative cohesion (*C_neg*), and total cohesion (*C_total*) were calculated referring to the scripts for HERREN and HERNANDEZ (HERREN, C M, et al., 2017; HERNANDEZ, D J, et al., 2021). The robustness for random species removal (*Robustness_R*), robustness for dominant species removal (*Robustness_Y*), and vulnerability metrics (*Vulnerability*) were calculated referring to the scripts for NEFF and YUAN (NEFF, F, et al., 2021; YUAN, M M, et al., 2021; GU, S, et al., 2023).

The indices of *C-diversity*, *C-phyl*, and *C-stability* were calculated according to the entropy weight method based on the indices of 1-8, 9-16, and 17-24 in **Sup Table 1**, respectively. The data processing process of entropy weight was as follows (ZHOU, K, 2022; PLIEGO-MARTÍNEZ, O, et al., 2024).

Step 1, data normalization.

$$\text{ }\text{X}\text{ }\text{=}\text{ }\left( \begin{matrix} \text{x}_{\text{11}} & \text{⋯} & \text{x}_{\text{1m}} \\ \text{⋮} & \text{⋱} & \text{⋮} \\ \text{x}_{\text{n1}} & \text{⋯} & \text{x}_{\text{nm}} \end{matrix} \right)\text{ }\text{ }\text{(}\text{x}_{\text{ij}}\text{, }\text{i}\text{=1, 2, 3, }\text{⋯}\text{, n j=1, 2, 3, }\text{⋯}\text{, m)}$$

where *n* is the number of samples, *m* is the number of indices in **Sup Table 1**, *x_ij_* is the value for the *j_th_* index of the *i_th_* sample.

$${\text{ }\text{y}}_{\text{ij}}\text{=}\left\{ \begin{aligned} \frac{\text{x}_{\text{ij}}\text{-min}\text{(X}_{\text{j}}\text{)}}{\text{max}\text{(X}_{\text{j}}\text{)-min}\text{(X}_{\text{j}}\text{)}}\text{ if }\text{X}_{\text{j}}\text{ belongs to positive indices } \\ \frac{\text{max}\text{(X}_{\text{j}}\text{)-}\text{x}_{\text{ij}}}{\text{max}\text{(X}_{\text{j}}\text{)-min}\text{(X}_{\text{m}}\text{)}}\text{ if }\text{X}_{\text{j}}\text{ belongs to negative indices} \end{aligned} \right.$$

where *y_ij_* is the value for the *j_th_* index of the *i_th_* sample.

Step 2, the determination of index entropy (CHENG, W, et al., 2020; LI, Q, et al., 2024).

$$\text{p}_{\text{ij}}\text{=}\frac{\text{y}_{\text{ij}}}{\sum_{\text{i}}^{\text{n}} \text{y}_{\text{ij}}}$$

$$\text{E}_{\text{j}}\text{ }\text{=}\text{ }\text{-}\frac{\text{1}}{\text{ln}\text{(}\text{n}\text{)}}\text{ }\sum_{\text{i=1}}^{\text{n}} \text{p}_{\text{ij}}\text{ln}\text{(}\text{p}_{\text{ij}}\text{)}$$

where *p_ij_* is the weight value of the *i_th_* sample in the *j_th_* index, and *E_j_* is the determination of the *j_th_* index entropy.

Step3, determine the weights of each index by the information redundancy.

$${\text{ }\text{W}}_{\text{j}}\text{ }\text{=}\text{ }\frac{\text{1-}\text{E}_{\text{j}}}{\sum\text{1-}\text{E}_{\text{j}}}$$

where *W_j_* is the weight of the *j_th_* index.

Step 4, the calculation of *C-diversity*, *C-phyl*, and *C-stability*.

$$\text{ }\text{C-diversity = }\sum_{\text{j=1}}^{\text{ m}} \text{W}_{\text{j}}\text{y}_{\text{ij}} \text{(j: indices of 1-8 in }\text{Sup }\text{T}\text{able}\text{ }\text{1}\text{)}$$

$$\text{ }\text{C-}\text{phyl}\text{ = }\sum_{\text{j=1}}^{\text{ m}} \text{W}_{\text{j}}\text{y}_{\text{ij}} \text{(j: indices of 9-16 in }\text{Sup}\text{ T}\text{able}\text{ }\text{1}\text{)}$$

$$\text{ }\text{C-stability = }\sum_{\text{j=1}}^{\text{ m}} \text{W}_{\text{j}}\text{y}_{\text{ij}} \text{(j: indices of 17-24 in }\text{Sup}\text{ T}\text{able}\text{ }\text{1}\text{)}$$

**B. The statements of network analysis for co-occurrence in Fig. 5 and Sup Fig. 5.**

The average degree (d), average path length (l), clustering coefficient (c), and modularity index (m) of the megabenthic community network in the estuary region during the spring dry period were 14.49, 1.78, 0.45, and 0.27, respectively (**Sup Fig. 5 IA-E**). There were 5 benthic dominant species in the estuary region, the main dominant species were *Leptochela gracilis* (*Y*=0.18) and *Philine otukai* (*Y*=0.17), and the abundance of *Philine otukai* reached 42.97% (**Sup Table 6**). During the spring dry period, the network complexity of the megabenthic community in the estuary region was low, and the community structure was relatively simple.

During the summer period before the WSRS, the network index of d, l, c, and m in the estuary region were 4.79, 3.17, 0.26, and 0.60, respectively (**Sup Fig. 5 IB-E**). There were 10 benthic dominant species in the estuary region, the main dominant species were *Chaeturichthys stigmatias* (*Y*=0.28), *Nassarius succinctus* (*Y*=0.19), and *Neverita didyma* (*Y*=0.11), and the abundance of *Chaeturichthys stigmatias* reached 31.95% (**Sup Table 6**). During this period, the biodiversity of megabenthos in the estuary was high, and the complexity of the community network was much higher than that in spring.

During the summer period after the WSRS, the network index of d, l, c, and m in the estuary region were 9.67, 2.46, 0.47, and 0.34, respectively (**Sup Fig. 5 IC-E**). Compared with the period before the WSRS, the potential interaction between species, network complexity, and community organization level in the estuary decreased drastically. There were 9 dominant species in the northern region of the estuary, those were *Chaeturichthys stigmatias* (*Y*=0.17), *Nassarius succinctus* (*Y*=0.08), *Oratosquilla oratoria* (*Y*=0.07), and others (**Sup Table 6**). There were 4 dominant species in the southern region of the estuary, those were *Modiolus elongatus* (*Y*=0.36), *Acetes chinensis* (*Y*=0.19), *Oratosquilla oratoria* (*Y*=0.07), and *Palaemon graviera* (*Y*=0.04). During the WSRS, influenced by the river flux input, two large faunas of megabenthos with different species composition, biodiversity, and community structure formed on the northern and southern sides of the estuary, respectively.

There were 5, 10, 9, and 4 dominant species (*Y* ≥ 0.02) that existed in the estuarine community of IA-E, IB-E, IC-EN, and IC-ES (**Sup Table 6**). The dominant taxa of megabenthos (family level) included Gobiidae, Cynoglossidae, Sciaenidae, Alpheidae, Pasiphaeidae, Squillidae, Palaemonidae, Nassariidae, Naticidae, Tellinidae, and Mytilidae (**Fig. 5**).

**C. The statements of seawater environmental parameters in Sup Table 5.**

At each environmental investigation site, samples of bottom seawater (2-3 meters above the seafloor) were collected using the CTD profiler (911 plus, Sea-bird Scientific, America) equipped with a 12 L Niskin bottle rosette sampler. The basic environmental parameters of sea temperature (Tem), salinity (Sa), pH, turbidity (Tu), depth, and chlorophyll *a* (Chl) were recorded by the CTD profiler. Seawater samples (0.2 L) were filtered *in situ* using 0.45-µm pore size acetate cellulose filters (47 mm diameter), and then immediately preserved at -20 ℃ refrigerator before laboratory analysis. Nutrient element concentrations, including seawater NO_3_^-^-N, NO_2_^-^-N, NH_4_^+^-N, PO_4_^3-^-P, and SiO_3_^2-^-Si, were measured in the laboratory using an autoanalyzer (AutoAnalyzer III, Seal Analytical, Germany). Dissolved inorganic nitrogen (DIN) primarily consists of NO_3_^-^-N, NH_4_^+^-N, and NO_2_^-^-N. The ratios of N/P, Si/N, and Si/P were calculated as the ratio of DIN to PO_4_^3-^-P, SiO_3_^2-^-Si to DIN, and SiO_3_^2-^-Si to PO_4_^3-^-P, respectively.

Compared with IA and IC, IB had the seawater characteristics of higher salinity, chlorophyll *a*, and lower turbidity, PO_4_^3-^-P, due to the influence of summer warming and the decrease of river flux input in the dry season. Moreover, compared with IA and IB, IC had the seawater characteristics of higher turbidity, pH, NH_4_^+^-N, NO_2_^-^-N, SiO_3_^2-^-Si, DSi/DIN, DSi/DIP, and lower NO_3_^-^-N, DIN, chlorophyll *a*, DIN/DIP, due to the significant increase of the river flux into the sea during the flood period. Therefore, the summer warming had a crucial impact on temperature, pH, chlorophyll *a*, PO_4_^3-^-P, and NO_3_^-^-N content of the bottom seawater in this area. While, the increase of flux into the sea during flood season dramatically changes the turbidity, chlorophyll *a*, nutrient contents, and nutrient structure of the bottom seawater in this area. In terms of spatial distribution characteristics, compared with the overall fluctuation of seawater parameters between IA and IB in the whole area, the distribution pattern of partial seawater parameters during flood season had an immense change. Influenced by the increase of river flux input and nearshore current, an extreme region of environmental condition (such as low salinity, high turbidity, high pH, and high SiO_3_^2-^-Si) has been formed from the Yellow River estuary to the west of the Laizhou Bay, leading a drastic shift of benthic habitat.

**Reference**

Cheng W, Xi H, Sindikubwabo C, et al., 2020. Ecosystem health assessment of desert nature reserve with entropy weight and fuzzy mathematics methods: A case study of Badain Jaran Desert[J]. Ecological Indicators, 119: 106843. DOI:10.1016/j.ecolind.2020.106843.

Faith D. 1992. Conservation evaluation and phylogenetic diversity[J]. Biological Conservation, 61(1): 1-10. DOI:10.1016/0006-3207(92)91201-3.

Gu S, Deng Y, Wang P, et al., 2023. Assessing riverine fish community diversity and stability by eDNA metabarcoding[J]. Ecological Indicators, 157: 111222. DOI:10.1016/j.ecolind.2023.111222.

Hernandez D, David A, Menges E S, et al., 2021. Environmental stress destabilizes microbial networks[J]. The ISME Journal, 15(6): 1722-1734. DOI:10.1038/s41396-020-00882-x.

Herren C, Mcmahon K. 2017. Cohesion: a method for quantifying the connectivity of microbial communities[J]. The ISME Journal, 11(11): 2426-2438. DOI:10.1038/ismej.2017.91.

Jiao W, Binduo X, Chongliang Z, et al., 2023. Effect of sampling design on estimation of phylogenetic diversity metrics of fish community[J]. Oecologia, 201(1): 129-141. DOI:10.1007/s00442-022-05291-9.

Li Q, Ma B, Zhao L, et al., 2024. Study on Spatial and Temporal Changes in Landscape Ecological Risks and Indicator Weights: A Case Study of the Bailong River Basin[J]. Sustainability, 16(5): 1915. DOI:10.3390/su16051915.

Margalef R. 1951. Diversidad de especies en las comunidades naturales[C]. Publicaciones del Instituto de Biologia Aplicada, 6(1): 59-72. http://hdl.handle.net/10261/165981.

Neff F, Brändle M, Ambarlı D, et al., 2021. Changes in plant-herbivore network structure and robustness along land-use intensity gradients in grasslands and forests[J]. Science Advances, 7(20): f3985. DOI:10.1126/sciadv.abf3985.

O W C. 2000. Exploring the Phylogenetic Structure of Ecological Communities: An Example for Rain Forest Trees[J]. The American naturalist, 156(2): 145-155. DOI:10.1086/303378.

Pielou E. 1966. The measurement of diversity in different types of biological collections[J]. Journal of Theoretical Biology, 13: 131-144. DOI: 10.1016/0022-5193(66)90013-0.

Pliego-Martínez O, Martínez-Rebollar A, Estrada-Esquivel H, et al., 2024. An Integrated Attribute-Weighting Method Based on PCA and Entropy: Case of Study Marginalized Areas in a City[J]. Applied Sciences, 14(5). DOI: 10.3390/app14052016.

R Helmus M, J Bland T, K Wiliams C, et al., 2007. Phylogenetic measures of biodiversity[J]. The American naturalist, 169(3): E68-E83. DOI:10.1086/511334.

Shannon C, Weaver W. 1949. The mathematical theory of communication[M]. Champaign, IL, US: University of Illinois Press: 117. DOI:10.1063/1.3067010.

Simpson E. 1949. Measurement of Diversity[J]. Nature, 163(4148): 688. DOI:10.1038/163688a0.

Teichert N, Lepage M, Chevillot X, et al., 2018. Environmental drivers of taxonomic, functional and phylogenetic diversity (alpha, beta and gamma components) in estuarine fish communities[J]. Journal of Biogeography, 45(2): 406-417. DOI:10.1111/jbi.13133.

Tilman D. 1999. The ecological consequences of changes in biodiversity: A search for general principles[J]. Ecology, 80(5): 1455-1474. DOI:10.1890/0012-9658(1999)080[1455:tecoci]2.0.co;2.

Weibing X, Yunpeng L, Wei L, et al., 2021. Specialized metabolic functions of keystone taxa sustain soil microbiome stability[J]. Microbiome, 9(1): 35. DOI:10.1186/s40168-020-00985-9.

Yuan M, Guo X, Wu L, et al., 2021. Climate warming enhances microbial network complexity and stability[J]. Nature Climate Change, 11(4): 343-348. DOI:10.1038/s41558-021-00989-9.

Zhou K. 2022. Comprehensive evaluation on water resources carrying capacity based on improved AGA-AHP method[J]. Applied Water Science, 12(5): 103. DOI:10.1007/s13201-022-01626-2.
